# Supplementary material for: Migration of Alpine Slavs and machine learning: Space-time pattern mining of an archaeological data set
Source: PLoS One. 2022 Sep 19;17(9):e0274687. doi: 10.1371/journal.pone.0274687 (PMC9484688; doi:10.1371/journal.pone.0274687)
Supplement: S1 Table — (DOCX) [file pone.0274687.s001.docx]

***S1 Table****: Archaeology-specific categories for archaeological trend map*.

| ID | Name | Description |
| --- | --- | --- |
| 1 | Persistent Hot Spot | A location has been a statistically significant hot spot for 90% of the time. |
| 2 | Sporadic Hot Spot | A location has been a statistically significant hot spot in every period, but less then 90% of the time in total. |
| 3 | Persistent Cold Spot | A location has been a statistically significant cold spot for 90% of the time. |
| 4 | Consecutive Cold Spot | A location with a single uninterrupted run of statistically significant cold spot in the final time-step intervals. |
| 5 | Sporadic Cold Spot | A location that is an on-again then off-again cold spot. Less than ninety percent of the time-step intervals have been statistically significant cold spots and none of the time-step intervals have been statistically significant hot spots. |
| 6 | Late Antiquity Persistent Hot Spot | A location has been a statistically significant hot spot for at least 200 years between 400 and 650. |
| 7 | Late Antiquity Consecutive Hot Spot | A location has been a statistically significant hot spot for at least 100 years between 400 and 650. |
| 8 | Late Antiquity and Early Middle Ages 1 Persistent Hot Spot | A location has been a statistically significant hot spot for at least 400 years between 400 and 900. |
| 9 | Late Antiquity and Early Middle Ages 1 Consecutive Hot Spot | A location has been a statistically significant hot spot for at least 200 years between 400 and 900 and hot spot before and after 650. |
| 10 | Early Middle Ages 1 Persistent Hot Spot | A location has been a statistically significant hot spot for at least 200 years between 650 and 900. |
| 11 | Early Middle Ages 1 Consecutive Hot Spot | A location has been a statistically significant hot spot for at least 100 years between 650 and 900. |
| 12 | Early Middle Ages 1 and 2 Persistent Hot Spot | A location has been a statistically significant hot spot for at least 400 years between 650 and 1100. |
| 13 | Early Middle Ages 1 and 2 Consecutive Hot Spot | A location has been a statistically significant hot spot for at least 200 years between 650 and 1100 and hot spot before and after 900. |
| 14 | Early Middle Ages 2 Persistent Hot Spot | A location has been a statistically significant hot spot for at least 150 years between 900 and 1100. |
| 15 | Early Middle Ages 2 Consecutive Hot Spot | A location has been a statistically significant hot spot for at least 100 years between 900 and 1100. |
| 16 | Late Antiquity and Early Middle Ages 2 Persistent Hot Spot | Hot spot for at least 200 years between 400 and 650 and for at least 150 years between 900 and 1100. |
