# Supplementary material for: Migration of Alpine Slavs and machine learning: Space-time pattern mining of an archaeological data set
Source: PLoS One. 2022 Sep 19;17(9):e0274687. doi: 10.1371/journal.pone.0274687 (PMC9484688; doi:10.1371/journal.pone.0274687)
Supplement: S1 Appendix — (DOCX) [file pone.0274687.s002.docx]

GIS Protocol for Multy-Scale Emerging Hot Spot Analysis

*Authors*

Benjamin Štular*^,a^, Edisa Lozić^a^

^a^ Znanstvenoraziskovalni center Slovenske akademije znanosti in umetnosti, Novi trg 2, SI-1000 Ljubljana, Slovenia.

* Corresponding author. Znanstvenoraziskovalni center Slovenske akademije znanosti in umetnosti, Novi trg 2, SI-1000 Ljubljana, Slovenia. *Email address:* [*benjamin.stular@zrc-sazu.si*](mailto:benjamin.stular@zrc-sazu.si).

# Abstract

This GIS protocol is primarily intended as supplementary material to the article (Štular et al., 2022). The article contains important contextual information about its intended use. In short, this GIS protocol was developed for the purposes of archaeological regional analysis of spatial data. The data are provided elsewhere in spreadsheet format (Štular et al., 2021). Data in GIS format are included in this repository. The GIS protocol can be used with any relevant data for any purpose as long as the data format matches the format of the included data.

# Keywords

Archaeology, GIS Protocol, Multy-Scale Emerging Hot Spot Analysis, Open Access

# 1. Multy-Scale Emerging Hot Spot Analysis

The multy-scale emerging hot spot analysis is a multi-step process executed entirely withinn the ArcGIS Pro 2.9 (ESRI, Redlands, CA, USA). This is proprietary commercial software sold under various pricing schemes, but a free trial is available. The entire process can be comfortably tested within the trial period (currently 7 days).


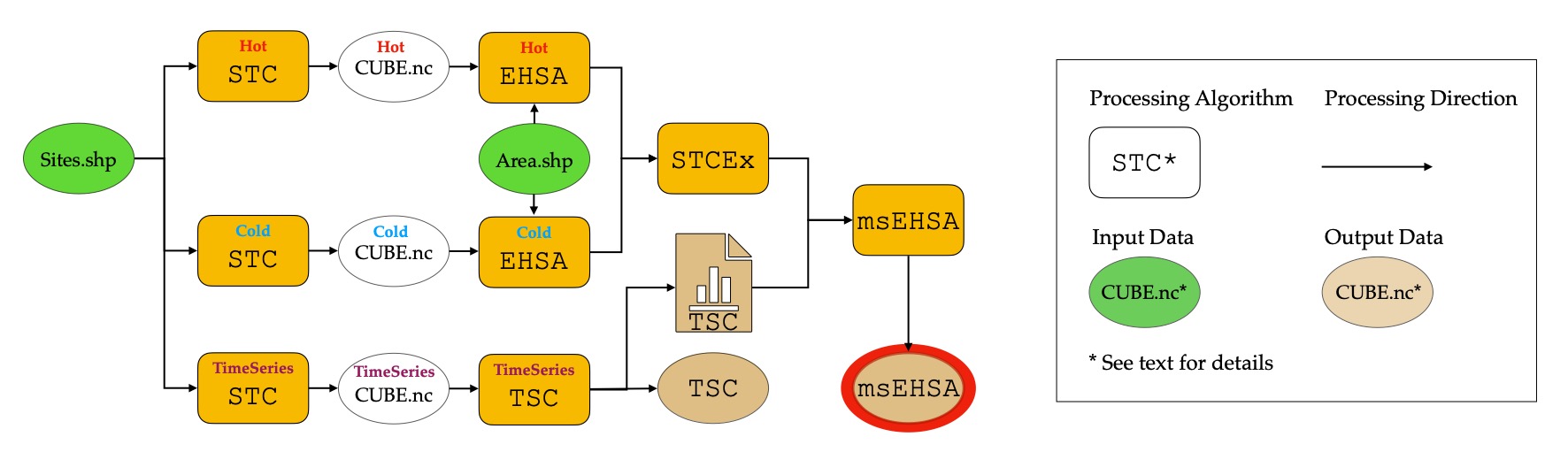
The process consists of six steps. The workflow pipeline is presented bellow in the form of flowchart:

Bellow, each step is described in detail following the GIS protocol scheme developed by (Forsyth et al., 2006).

# 2. Create a Space Time Cube by Aggregating Points (STC)

## 2.1. Basic Concept

Create space-time cube by aggregating points summarizes a set of points in a netCDF data structure by aggregating them into space-time bins. Within each bin, the points are counted, and the specified attributes are aggregated. For all bin locations, the trend for the counts and summary field values are evaluated (ESRI, 2021a).

## 2.2. Basic Procedure

An automated process with a graphical user interface. The user provides the input data and specifies the parameters. The settings and hardware used in our analysis are listed below. All data marked with an asterisk* are available in an open-access Zenodo repository (Lozić, Štular 2022) in exactly the same form as used in our experiment.

| Space-Time Cube (Cold Spots) | | |
| --- | --- | --- |
| Software | ArcGIS Pro 2.9, ESRI, Redlands, CA, USA | |
| Hardware | MacBook Pro 16" (2020), running Windows 10 as a virual machine on Parallels 16 | |
| Tool | Create Space Time Cube By Aggregating Points | |
| Parameters | Input features: *__Sites2022_v01.shp** | |
|  | Output Space Time Cube: *CUBE_2022v1b_Cold_SiteValue.nc* | |
|  | Time Field: *Date2* | |
|  | Template Cube: *Leave Empty* | |
|  | Time Step Interval: *25 Years* | |
|  | Aggregation Shape Type: *Hexagon grid* | |
|  | Distance Interval: *5 Kilometers* | |
|  | Location ID: *ID2* | |
|  | Summary Fields | |
|  |  | Field: *sitevalue* |
|  |  | Statistic: *Sum* |
|  |  | Fill Empty Bins with: *Zeros* |
| Space-Time Cube (Hot Spots) | | |
| Software | ArcGIS Pro 2.9, ESRI, Redlands, CA, USA | |
| Hardware | MacBook Pro 16" (2020), running Windows 10 as a virual machine on Parallels 16 | |
| Tool | Create Space Time Cube by Aggregating Points | |
| Parameters | Input features: *__Sites2022_v01.shp** | |
|  | Output Space Time Cube: *CUBE_2022v1b_Hot_SiteValue.nc* | |
|  | Time Field: *Date2* | |
|  | Template Cube: *Leave Empty* | |
|  | Time Step Interval: *25 Years* | |
|  | Aggregation Shape Type: *Hexagon grid* | |
|  | Distance Interval: *5 Kilometers* | |
|  | Location ID: *ID2* | |
|  | Summary Fields | |
|  |  | Field: *sitevalue* |
|  |  | Statistic: *Sum* |
|  |  | Fill Empty Bins with: *Zeros* |

## 2.3. Detailed Definition

See ESRI 2021, under title »Create Space Time Cube By Aggregating Points (Space Time Pattern Mining)« (available online at <https://pro.arcgis.com/en/pro-app/latest/tool-reference/space-time-pattern-mining/create-space-time-cube.htm>, last checked on 2 January 2022).

## 2.4. Comments and Explanations

Due to the way ArcGIS Pro and especially the Space Time Cube Explorer add-on handle netCDF data, separate time cubes must be created for each analysis. In our particular example, i.e. for the creation of the multi-scale emerging hot spot analysis, two separate time cubes are required, one for cold spots and one for hot spots.

## 2.5. GIS Approach

The GIS approach involves using the properly formated sites data in shape (*.shp) format to produce the netCDF data structure in netCDF format (*.nc).

## 2.6. GIS Steps

This is a single step automated procedure. To see how it fits in the analysis process, see above (1. Multy-Scale Emerging Hot Spot Analysis).

# 3. Emerging Hot Spot Analysis (EHSA)

## 3.1. Basic Concept

Emerging Hot Spot Analysis (hereafter EHSA) identifies trends in the clustering of point densities (counts) or values in a space-time cube created using either the Create Space Time Cube By Aggregating Points, Create Space Time Cube From Defined Locations, or Create Space Time Cube from Multidimensional Raster Layer tool (ESRI, 2021a).

## 3.2. Basic Procedure

An automated process with a graphical user interface. The user provides the input data and specifies the parameters. The settings and hardware used in our analysis are listed below. All data marked with an asterisk* are available in an open-access Zenodo repository (Lozić, Štular 2022) in exactly the same form as used in our experiment.

| Emerging Hot Spot Analysis (Cold Spots) | |
| --- | --- |
| Software | ArcGIS Pro 2.9, ESRI, Redlands, CA, USA |
| Hardware | MacBook Pro 16" (2020), running Windows 10 as a virual machine on Parallels 16 |
| Tool | Emerging Hot Spot Analysis |
| Parameters | Input Space Time Cube: *CUBE_2022v1b_Cold_SiteValue.nc* |
|  | Output Features: *2022v0b_EHSA_Sitevalue_Cold.shp* |
|  | Conceptualization of Spatial Relationships: *Fixed Distance* |
|  | Neighborhood Distance: *20 Kilometres* |
|  | Number of Spatial Neighbors: *8* |
|  | Neighborhood Time Step: *3* |
|  | Polygon Analysis Mask: *Area1401.shp** |
|  | Define Global Window: *Entire Cube* |
| Emerging Hot Spot Analysis (Hot Spots) | |
| Software | ArcGIS Pro 2.9, ESRI, Redlands, CA, USA |
| Hardware | MacBook Pro 16" (2020), running Windows 10 as a virual machine on Parallels 16 |
| Tool | Create Space Time Cube By Aggregating points |
| Parameters | Input Space Time Cube: *CUBE_2022v1b_Hot_SiteValue.nc* |
|  | Output Features: *2022v0b_EHSA_Sitevalue_Hot.shp* |
|  | Conceptualization of Spatial Relationships: *K nearest neighbors* |
|  | Number of Spatial Neighbors: 6 |
|  | Neighborhood Time Step: 1 |
|  | Polygon Analysis Mask: *Area1401.shp** |
|  | Define Global Window: *Entire Cube* |

## 3.3. Detailed Definition

See ESRI 2021, under the title »Emerging Hot Spot Analysis (Space Time Pattern Mining)« (available online at https://pro.arcgis.com/en/pro-app/latest/tool-reference/space-time-pattern-mining/emerginghotspots.htm, last checked 2 January 2022).

## 3.4. Comments and Explanations

Because of the way ArcGIS Pro and especially the Space Time Cube Explorer add-on handle netCDF data, separate emerging hot spot analysis must be performed for hot and cold using separate time cubes as input to create the multi-scale emerging hot spot analysis.

## 3.5. GIS Approach

The GIS approach involves using the properly formatted netCDF data structure in netCDF format (*.nc) to create the File geodatabase feature class. The data relevant to the next steps of the process are written into the existing netCDF data structure in netCDF format (*.nc).

## 3.6. GIS Steps

This is a single step automated procedure. To see how it fits in the analysis process, see above (1. Multy-Scale Emerging Hot Spot Analysis).

# 4. Space-Time Cube Explorer – Create and Visualize (STCEx)

## 4.1. Basic Concept

With the Space Time Cube Explorer, you can visualize and explore your 3D Space Time Pattern Mining analysis results fast and easy. This Add-in uses your space-time cube as input and creates layers that can be visualized in a number of useful ways (ESRI, 2021b).

## 4.2. Basic Procedure

An automated process with a graphical user interface. The user provides the input data and specifies the parameters. The settings and hardware used in our analysis are listed below.

| Emerging Hot Spot Analysis (Cold Spots) | |
| --- | --- |
| Software | ArcGIS Pro 2.9 - Space Time Cube Explorer Add-in for ArcGIS Pro 2.9, ESRI, Redlands, CA, USA |
| Hardware | MacBook Pro 16" (2020), running Windows 10 as a virual machine on Parallels 16 |
| Tool | Space-Time Cube Explorer Add-in |
| Parameters | Create Layer - Input Cube: *CUBE_2022v1b_Cold_SiteValue.nc* |
|  | Create Layer - Output Feature Class: *_Explorer_2022b_Cold* |
|  | Create Layer - Analysis Type: *Emerging Hot Spot* |
|  | Visualize Layer – Select Layer: *Emerging Hot Spot* |
|  | Visualize Layer – Select Cube Variable: *SITEVALUE_SUM_ZERO* |
|  | Visualize Layer – Symbol Size: *Real-world* |
|  | Display Theme Gallery – Color By Hot Spot Significance: *All content* |
| Emerging Hot Spot Analysis (Hot Spots) | |
| Software | ArcGIS Pro 2.9, ESRI, Redlands, CA, USA |
| Hardware | MacBook Pro 16" (2020), running Windows 10 as a virual machine on Parallels 16 |
| Tool | Create Space Time Cube By Aggregating points |
| Parameters | Create Layer - Input Cube: *CUBE_2022v1b_Hot_SiteValue.nc* |
|  | Create Layer - Output Feature Class: *_Explorer_2022b_Hot* |
|  | Create Layer - Analysis Type: *Emerging Hot Spot* |
|  | Visualize Layer – Select Layer: *Emerging Hot Spot* |
|  | Visualize Layer – Select Cube Variable: *SITEVALUE_SUM_ZERO* |
|  | Visualize Layer – Symbol Size: *Real-world* |

## 4.3. Detailed Definition

See (ESRI, 2021b), under the title »Space-Time Cube Explorer Add-in for ArcGIS Pro 2.8« (available online at https://spatialstats-analysis-1.hub.arcgis.com/pages/space-time-cube-explorer, last checked on 2 January 2022).

## 4.4. Comments and Explanations

Because of the way ArcGIS Pro and especially the Space Time Cube Explorer add-on handle netCDF data, separate emerging hot spot analysis must be performed for hot and cold using separate time cubes as input to create the multi-scale emerging hot spot analysis.

## 4.5. GIS Approach

The GIS approach involves using the properly formatted netCDF data structure in netCDF format (*.nc) to create the File geodatabase feature class, which can be visualized as any other data in the ArcGIS Pro environment.

## 4.6. GIS Steps

This is a one-step automated procedure. The only goal of this step is to visualize the data. Unfortunately, ArcGIS Pro does not allow you to export individual time slice data. To see how this step fits into the analysis process, see above (1. Multy-Scale Emerging Hot Spot Analysis).

# 5. Time Series Clustering (TSC)

## 5.1. Basic Concept

The Time Series Clustering tool identifies the locations in a space-time cube that are most similar and partitions them into distinct clusters in which members of each cluster have similar time series characteristics (ESRI, 2021b).

## 5.2. Basic Procedure

An automated process with a graphical user interface. The user provides the input data and specifies the parameters. The settings and hardware used in our analysis are listed below. All data marked with an asterisk* are available in an open-access Zenodo repository (Lozić, Štular 2022) in exactly the same form as used in our experiment. Space Time Cube marked with double asterisk** must be created with the »Create Space Time Cube From Defined Locations« using the same parameters as described above (2.2: Hot Spots).

| Time Series Clustering | |
| --- | --- |
| Software | ArcGIS Pro 2.9, ESRI, Redlands, CA, USA |
| Hardware | MacBook Pro 16" (2020), running Windows 10 as a virual machine on Parallels 16 |
| Tool | Time Series Clustering |
| Parameters | Input Space-Time Cube: *CUBE_2022v1b_Point_SiteValue.nc*^,^*** |
|  | Analysis Variable: *COUNT* |
|  | Output Features: *TimeSeriesClustering_ProfileF.shp* |
|  | Characteristic of Interest: *Profile (Fourier)* |
|  | Time Leg: *CHECK*; Range: *UNCHECK* |
|  | Number of Clusters: *3* |
|  | Output Table for Charts: *Table_TimeSeriesClustering_ProfileF* |

## 5.3. Detailed Definition

See (ESRI, 2021b), under the title »Space Time Cube Explorer Add-in for ArcGIS Pro 2.8« (available online at https://pro.arcgis.com/en/pro-app/latest/tool-reference/space-time-pattern-mining/learnmoretimeseriesclustering.htm, last checked 2 January 2022).

## 5.4. Comments and Explanations

Because of the way ArcGIS Pro handle netCDF data, time cube must be created.

## 5.5. GIS Approach

The GIS approach involves using the properly formatted netCDF data structure in netCDF format (*.nc) to create the vector point data, which can be visualized as any other data in the ArcGIS Pro environment. In addition, table data are created, which can be visualized in the ArcGIS Pro environment or exported as data or image.

## 5.6. GIS Steps

This is a one-step automated procedure. The goal of this step is to create and visualize data in GIS and table format. The created data are, to a large degree, software agnostic.

# 6. Multy-Scale Emerging Hot Spot Analysis (msEHSA)

## 6.1. Basic Concept

This is a custome manual process developed specifically for our analysis. To see how this step fits into the analysis process, see above (1. Multy-Scale Emerging Hot Spot Analysis).

## 6.2. Basic Procedure

This is a manual process with a graphical user interface. Because ArcGIS Pro does not allow you to export individual time slice data, the results provided in Step 4 (STCEx) must be evaluated and input manually. The polygon grid is copied, e.g., from *_Explorer_2022b_Hot*, and new values are input according to the multi-scale emerging hot spot analysis key provided bellow:

| ID | Name | Description |
| --- | --- | --- |
| 1 | Persistent Hot Spot | A location has been a statistically significant hot spot for 90% of the time. |
| 2 | Sporadic Hot Spot | A location has been a statistically significant hot spot in every period, but less then 90% of the time in total. |
| 3 | Persistent Cold Spot | A location has been a statistically significant cold spot for 90% of the time. |
| 4 | Consecutive Cold Spot | A location with a single uninterrupted run of statistically significant cold spot in the final time-step intervals. |
| 5 | Sporadic Cold Spot | A location that is an on-again then off-again cold spot. Less than ninety percent of the time-step intervals have been statistically significant cold spots and none of the time-step intervals have been statistically significant hot spots. |
| 6 | Late Antiquity Persistent Hot Spot | A location has been a statistically significant hot spot for at least 200 years between 400 and 650. |
| 7 | Late Antiquity Consecutive Hot Spot | A location has been a statistically significant hot spot for at least 100 years between 400 and 650. |
| 8 | Late Antiquity and Early Middle Ages 1 Persistent Hot Spot | A location has been a statistically significant hot spot for at least 400 years between 400 and 900. |
| 9 | Late Antiquity and Early Middle Ages 1 Consecutive Hot Spot | A location has been a statistically significant hot spot for at least 200 years between 400 and 900 and hot spot before and after 650. |
| 10 | Early Middle Ages 1 Persistent Hot Spot | A location has been a statistically significant hot spot for at least 200 years between 650 and 900. |
| 11 | Early Middle Ages 1 Consecutive Hot Spot | A location has been a statistically significant hot spot for at least 100 years between 650 and 900. |
| 12 | Early Middle Ages 1 and 2 Persistent Hot Spot | A location has been a statistically significant hot spot for at least 400 years between 650 and 1100. |
| 13 | Early Middle Ages 1 and 2 Consecutive Hot Spot | A location has been a statistically significant hot spot for at least 200 years between 650 and 1100 and hot spot before and after 900. |
| 14 | Early Middle Ages 2 Persistent Hot Spot | A location has been a statistically significant hot spot for at least 150 years between 900 and 1100. |
| 15 | Early Middle Ages 2 Consecutive Hot Spot | A location has been a statistically significant hot spot for at least 100 years between 900 and 1100. |
| 16 | Late Antiquity and Early Middle Ages 2 Persistent Hot Spot | Hot spot for at least 200 years between 400 and 650 and for at least 150 years between 900 and 1100. |

## 6.3. Detailed Definition

msEHSA is modified trend map provided by 3. EHSA created by focusing on chronological periods calculated by the time series clustering method created in Step 5 (TSC). For example, a location was considered a first period consecutive hot spot if it had an uninterrupted hot spot series of at least 100 years within the first period. We term this an “archaeological trend map”.

In emerging hot spot analysis, the spatial and temporal neighborhoods have a significant influence on the results. We found, through empirical observation, that the best results for hot spots and cold spots were obtained with different settings: (i) fixed distance method with 20 km neighborhood, and time step three for cold spots; (ii) k-nearest neighbors (kNN) method with six spatial neighbors, time step one for hot spots.

Therefore, we have introduced another archaeology-specific modification to the tool. For the purposes of this article, we superimposed the cold spots derived using the first calculation (i) with the hot spots derived using the second calculation (ii) in a single visualization. We refer to this method as 'multiscale emerging hot spot analysis'.

## 6.4. Comments and Explanations

Right now, this is a knowledge-based manual GIS-assisted process. The process could easily be automated if ESRI offered to export time-slice data. At the moment, there are no plans to automate the process.

## 6.5. GIS Approach

The GIS approach involves visual exploration of the 3. EHSA data to manually create the polygon vector data file (e.g. shp), which can then be visualised and/or further analysed. The process is GIS-software independent.

## 6.6. GIS Steps

This is a one-step manual procedure. The only goal of this step is to create vector data. To see how this step fits into the analysis process, see above (1. Multy-Scale Emerging Hot Spot Analysis).

# 7. References

ESRI. (2021a). How Create Space Time Cube works. *ESRI Help*.

ESRI. (2021b). *Space Time Cube Explorer Add-in for ArcGIS Pro 2.8*.

Forsyth, A., Schmitz, K. H., Oakes, M., Zimmerman, J., & Koepp, J. (2006). Standards for Environmental Measurement Using GIS: Toward a Protocol for Protocols. *Journal of Physical Activity and Health*, *3*(s1), S241–S257. https://doi.org/10.1123/jpah.3.s1.s241

Štular, B., Lozić, E., Belak, M., Rihter, J., Koch, I., Modrijan, Z., Magdič, A., Karl, S., Lehner, M., & Gutjahr, C. (Submitted). *Migration of Alpine Slavs and Machine Learning: Space-Time Pattern Mining of an Early Medieval Data Set from the Eastern Alps*.

Štular, B., Pleterski, A., & Belak, M. (2021). *Zbiva, Early Medieval Data Set for the Eastern Alps. Data sub-set*. https://doi.org/10.5281/ZENODO.5761811
