# Supplementary figures and images for: Migration of Alpine Slavs and machine learning: Space-time pattern mining of an archaeological data set
Source: PLoS One. 2022 Sep 19;17(9):e0274687. doi: 10.1371/journal.pone.0274687 (PMC9484688; doi:10.1371/journal.pone.0274687)

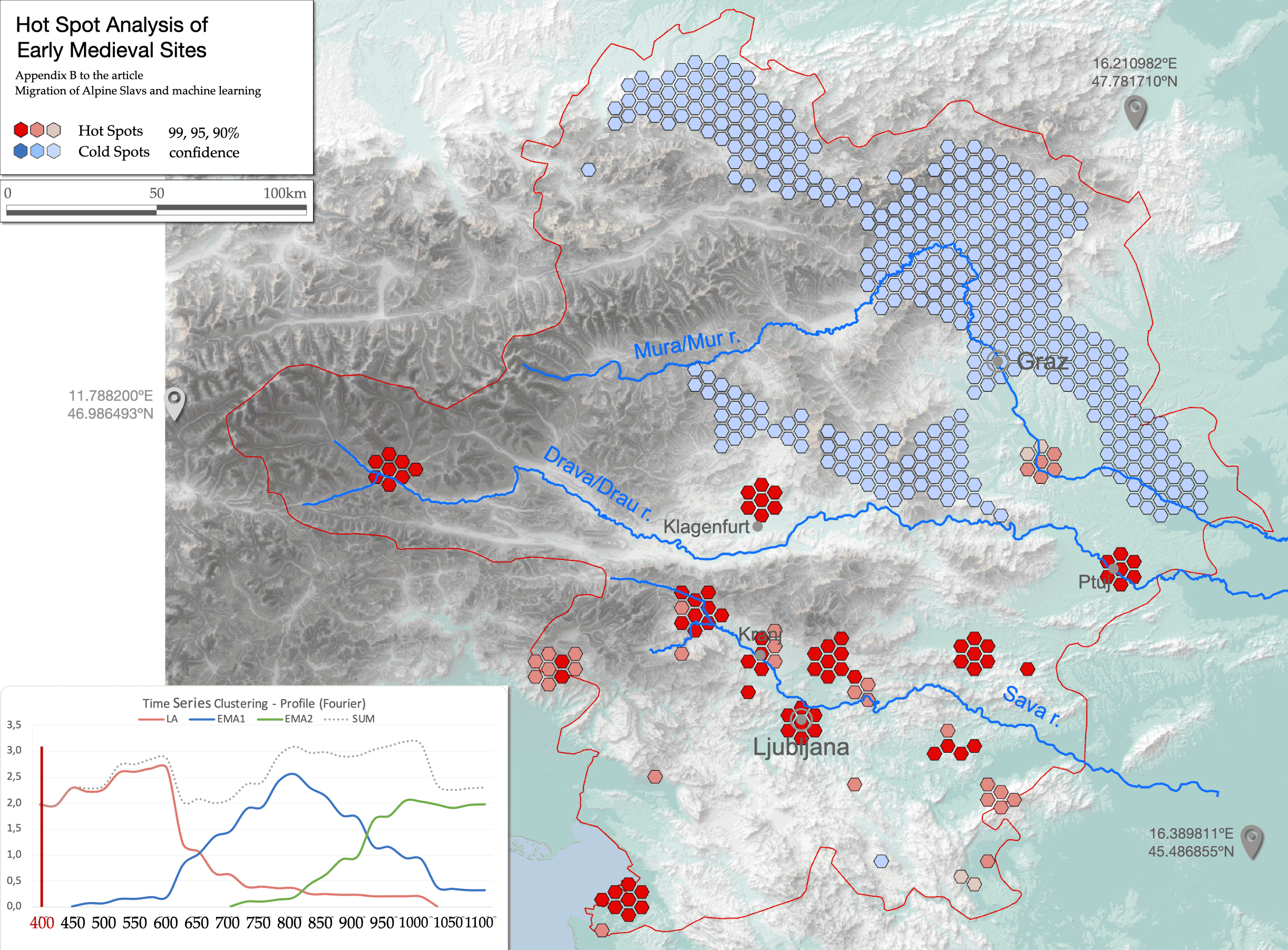

Supplement: S2 Appendix — (GIF) [file pone.0274687.s003.gif]
