# Supplementary material for: Migration of Alpine Slavs and machine learning: Space-time pattern mining of an archaeological data set
Source: PLoS One. 2022 Sep 19;17(9):e0274687. doi: 10.1371/journal.pone.0274687 (PMC9484688; doi:10.1371/journal.pone.0274687)
Supplement: Attachment — Submitted filename: PONE-D-22-00071_reviewer - comments.pdf [file pone.0274687.s004.pdf]

PLOS ONE

Migration of Alpine Slavs and Machine Learning: Space-Time Pattern Mining of an Early Medieval Data Set from the Eastern Alps

--Manuscript Draft--

**Manuscript Number:**	PONE-D-22-00071
**Article Type:**	Research Article
**Full Title:**	Migration of Alpine Slavs and Machine Learning: Space-Time Pattern Mining of an Early Medieval Data Set from the Eastern Alps
**Short Title:**	Migration of Alpine Slavs and Machine Learning
**Corresponding Author:**	Benjamin Štular, Ph.D.<br>Znanstvenoraziskovalni center Slovenske akademije znanosti in umetnosti<br>Ljubljana, SLOVENIA
**Keywords:**	Early Medieval Period; Early Middle Ages; migration of Slavs; machine learning; space-time pattern mining; Deep Data; Alpine Slavs
**Abstract:**	The rapid expansion of the Slavic language in the second half of the first millennium CE remains a controversial topic, and academic passions on the issue have long run high. Currently, there are three main hypotheses for this expansion. The aim of this paper was to test the so-called “hybrid hypothesis,” which states that the movement of people, cultural diffusion and language diffusion all occurred simultaneously. For this purpose, we examined an archaeological Deep Data set with a machine learning method time series clustering and with emerging hot spot analysis. The latter required two archaeology-specific modifications: The archaeological trend map and the multiscale emerging hot spot analysis. As a result, we were able to detect two migrations in the Eastern Alps between c. 500 and c. 700 CE. Based on the convergence of evidence from archaeology, linguistics, and population genetics, we have identified the migrants as Alpine Slavs, i.e., people who spoke Common Slavic and shared specific ancestry, which was in common to them and is in common to modern speakers of Slavic language.
**Order of Authors:**	Benjamin Štular, Ph.D.
	Edisa Lozić
	Mateja Belak
	Jernej Rihter
	Iris Koch
	Zvezdana Modrijan
	Andrej Magdič
	Stephan Karl
	Manfred Lehner
	Christoph Gutjahr
**Additional Information:**	
**Question**	**Response**
**Financial Disclosure**<br><br>Enter a financial disclosure statement that describes the sources of funding for the work included in this submission. Review the submission guidelines for detailed requirements. View published research articles from *PLOS ONE* for specific	The funders had no role in study design, data collection and analysis, decision to publish, or preparation of the manuscript.

Powered by Editorial Manager® and ProduXion Manager® from Aries Systems Corporation

examples.<br><br>This statement is required for submission and **will appear in the published article** if the submission is accepted. Please make sure it is accurate.<br><br>**Unfunded studies**<br>Enter: *The author(s) received no specific funding for this work.*<br><br>**Funded studies**<br>Enter a statement with the following details:<br>• Initials of the authors who received each award<br>• Grant numbers awarded to each author<br>• The full name of each funder<br>• URL of each funder website<br>• Did the sponsors or funders play any role in the study design, data collection and analysis, decision to publish, or preparation of the manuscript?<br>• **NO** - Include this sentence at the end of your statement: *The funders had no role in study design, data collection and analysis, decision to publish, or preparation of the manuscript.*<br>• **YES** - Specify the role(s) played.<br><br>* typeset	
**Competing Interests**<br><br>Use the instructions below to enter a competing interest statement for this submission. On behalf of all authors, disclose any competing interests that could be perceived to bias this work—acknowledging all financial support and any other relevant financial or non-financial competing interests.<br><br>This statement is required for submission and **will appear in the published article** if the submission is accepted. Please make sure it is accurate and that any funding sources listed in your Funding Information later in the submission form are also declared in your Financial Disclosure statement.	The authors have declared that no competing interests exist.

View published research articles from *PLOS ONE* for specific examples.<br><br>**NO authors have competing interests**<br><br>Enter: *The authors have declared that no competing interests exist.*<br><br>**Authors with competing interests**<br><br>Enter competing interest details beginning with this statement:<br><br>*I have read the journal's policy and the authors of this manuscript have the following competing interests: [insert competing interests here]*<br><br>* typeset	
**Ethics Statement**<br><br>Enter an ethics statement for this submission. This statement is required if the study involved:<br><br>• Human participants<br>• Human specimens or tissue<br>• Vertebrate animals or cephalopods<br>• Vertebrate embryos or tissues<br>• Field research<br><br>Write "N/A" if the submission does not require an ethics statement.<br><br>General guidance is provided below. Consult the submission guidelines for detailed instructions. **Make sure that all information entered here is included in the Methods section of the manuscript.**	N/A

**Format for specific study types**<br><br>**Human Subject Research (involving human participants and/or tissue)**<br>• Give the name of the institutional review board or ethics committee that approved the study<br>• Include the approval number and/or a statement indicating approval of this research<br>• Indicate the form of consent obtained (written/oral) or the reason that consent was not obtained (e.g. the data were analyzed anonymously)<br><br>**Animal Research (involving vertebrate animals, embryos or tissues)**<br>• Provide the name of the Institutional Animal Care and Use Committee (IACUC) or other relevant ethics board that reviewed the study protocol, and indicate whether they approved this research or granted a formal waiver of ethical approval<br>• Include an approval number if one was obtained<br>• If the study involved *non-human primates*, add *additional details* about animal welfare and steps taken to ameliorate suffering<br>• If anesthesia, euthanasia, or any kind of animal sacrifice is part of the study, include briefly which substances and/or methods were applied<br><br>**Field Research**<br><br>Include the following details if this study involves the collection of plant, animal, or other materials from a natural setting:<br>• Field permit number<br>• Name of the institution or relevant body that granted permission	
**Data Availability**<br><br>Authors are required to make all data underlying the findings described fully available, without restriction, and from the time of publication. PLOS allows rare exceptions to address legal and ethical concerns. See the PLOS Data Policy and FAQ for detailed information.	Yes - all data are fully available without restriction

Powered by Editorial Manager® and ProduXion Manager® from Aries Systems Corporation

A Data Availability Statement describing where the data can be found is required at submission. Your answers to this question constitute the Data Availability Statement and **will be published in the article**, if accepted.<br><br>**Important:** Stating 'data available on request from the author' is not sufficient. If your data are only available upon request, select 'No' for the first question and explain your exceptional situation in the text box.<br><br>Do the authors confirm that all data underlying the findings described in their manuscript are fully available without restriction?	
**Describe where the data may be found in full sentences. If you are copying our sample text, replace any instances of XXX with the appropriate details.**<br><br>• If the data are **held or will be held in a public repository**, include URLs, accession numbers or DOIs. If this information will only be available after acceptance, indicate this by ticking the box below. For example: *All XXX files are available from the XXX database (accession number(s) XXX, XXX.).*<br>• If the data are all contained **within the manuscript and/or Supporting Information files**, enter the following: *All relevant data are within the manuscript and its Supporting Information files.*<br>• If neither of these applies but you are able to provide **details of access elsewhere**, with or without limitations, please do so. For example:<br><br>*Data cannot be shared publicly because of [XXX]. Data are available from the XXX Institutional Data Access / Ethics Committee (contact via XXX) for researchers who meet the criteria for access to confidential data.*<br><br>*The data underlying the results presented in the study are available from (include the name of the third party*	All relevant files are available from the Zenodo database at https://zenodo.org/record/5813527 (doi: 10.5281/zenodo.5813527) and https://zenodo.org/record/5761811 (doi: 10.5281/ZENODO.5761811).

and contact information or URL).<br>• This text is appropriate if the data are owned by a third party and authors do not have permission to share the data.<br><br>* typeset	
Additional data availability information:	

Powered by Editorial Manager® and ProduXion Manager® from Aries Systems Corporation

Manuscript

Click here to access/download;Manuscript;StularEt_Manuscript.docx

Full title

Migration of Alpine Slavs and machine learning: space-time pattern mining of an Early Medieval data set from the Eastern Alps

Short title

Migration of Alpine Slavs and machine learning

Keywords: Early Medieval Period, Early Middle Ages, migration of Slavs, machine learning, space-time pattern mining, Deep Data, Alpine Slavs.

Authors

Benjamin Štular*,[a], Edisa Lozić[a,b], Mateja Belak[a], Jernej Rihter[a], Iris Koch[b], Zvezdana Modrijan[a], Andrej Magdič[c], Stephan Karl[b], Manfred Lehner[b], Christoph Gutjahr[b]

[a] Znanstvenoraziskovalni center Slovenske akademije znanosti in umetnosti, Novi trg 2, SI-1000 Ljubljana, Slovenia.

[b] Institute of Classics, University of Graz, 8010 Graz, Austria

[c] Javni zavod Republike Slovenije za varstvo kulturne dediščine, Slomškov trg 6, SI-2000 Maribor, Slovenia.

* Corresponding author. Znanstvenoraziskovalni center Slovenske akademije znanosti in umetnosti, Novi trg 2, SI-1000 Ljubljana, Slovenia. *Email address: : benjamin.stular@zrc-sazu.si*.

Abstract

The rapid expansion of the Slavic language in the second half of the first millennium CE remains a controversial topic, and academic passions on the issue have long run high. Currently, there are three main hypotheses for this expansion. The aim of this paper was to test the so-called “hybrid hypothesis,” which states that the movement of people, cultural diffusion and language diffusion all occurred simultaneously. For this purpose, we examined an archaeological Deep Data set with a machine learning method time series clustering and with emerging hot spot analysis. The latter required two archaeology-specific modifications: The archaeological trend map and the multiscale emerging hot spot analysis. As a result, we were able to detect two migrations in the Eastern Alps between c. 500 and c. 700 CE. Based on the convergence of evidence from archaeology, linguistics, and population genetics, we have identified the migrants as Alpine Slavs, i.e., people who spoke Common Slavic and shared specific ancestry, which was in common to them and is in common to modern speakers of Slavic language.

1. Introduction

On Easter Monday, 2 April 568 CE, Alboin, son of Audoin and king of the Lombards, led his people on an arduous journey. They were to “abandon the barren fields...and come and take possession of Italy." Because from the "west and north [Italy] is shut in by the range of Alps, they reached it from the eastern side by which it is joined to Pannonia” (Paul the Deacon, Book II, Ch. V. and IX.; [1].

Historiography records Alboin's success: He founded the Kingdom of Italy the next year and his successors ruled there for almost two centuries, e.g., [2]. However, historiography is less clear about events in the lands that Alboin and his people left behind. These lands include the region we know as the Eastern Alps; we know little more than that this region was sparsely populated by Romans in the sixth century and that several peoples, e.g. Ostrogoths, Gepids, and Slavs,

attempted to make it their home, e.g., [3,4]. Of these peoples, the Slavs were the most successful.

The rapid spread of the Slavic language in the second half of the first millennium CE remains a controversial topic. There are two main reasons for this. First, the lack of first-hand, written sources before the end of the ninth century. Unlike the Lombards, the Slavs had no Paul the Deacon to recount their early history. Second, archaeological evidence on this subject is sparse compared to many other Early Medieval peoples. As a result, there is a "propensity for sweeping explanations" [5].

Currently, there are three main hypotheses for the spread of the Common Slavic idiom, as linguists define the structure of a language spoken between about 400 and 850 CE, e.g., [6,7]. The first hypothesis assumes that speakers moved in all directions from their small original habitat, the so-called *Urheimat*, e.g., [8–10]. The second hypothesis assumes the diffusion of the Slavic cultural model among non-Slavic populations or, in its extreme form, the diffusion of language alone, e.g., [7,11–14]. Many archaeologists adhere to the third, hybrid hypothesis. The hybrid hypothesis states that movement, cultural diffusion, and language diffusion occurred simultaneously [15–18]. This is supported by recent research in population genetics and linguistics. It seems that the language spread in the West Slavic zone mainly by migration to sparsely populated areas, and in the East Slavic zone by a combination of migration and language shift. The spread in the South Slavic region was triggered by migration, but the main mechanism for further spread was a language shift from local Balkan idioms to Slavic [19].

We adhere to the hybrid hypothesis in its most recent form [19], which is based primarily on population genetics and language studies. The aim of this paper was to test this hypothesis with archaeological data from the Eastern Alps. The specific objective of the paper was to elucidate the settlement of the Alpine Slavs, as the Common Slavic-speaking Early Mediaeval population of the Eastern Alps is known in historiography [4,20–22]. To this end, we applied a machine learning technique called “space-time pattern mining” to examine a large archaeological data set

from the period 400 to 1100 CE. The methods we developed~~,~~ can be applied to archaeological studies of any period, anywhere that sufficient data is available.

2. Material and Methods

2.1. Data Set and Study Area

Our data set is Zbiva [23], which is an open access research database for the archaeology of the Eastern Alps in the Early Middle Ages (in our study 600 to 1100 CE). The inception of the database in the early 1980s was deeply rooted in the scientific research context of that time, which determined both the geographical and chronological focus of the data set [24].

Currently, Zbiva contains data on 3,379 sites and 11.596 related bibliographic units in more than half a million database fields. Because the data set is the result of four decades of deliberate scholarly work and attentive curation, it is best described by the concept of Deep Data. The Deep Data approach is one in which we make full use of all the information available in the data to gain ~~the~~ knowledge [25], *cf.* [26]. As far as the authors are aware, Zbiva is unmatched in Slavic archaeology, and the only comparable data set for Early Medieval archaeology is OpenAtlas [27], with its affiliate, THANADOS [28].

Recently, the team behind Zbiva has enriched the database, adding new information with a focus on chronology and location. The chronology of each site was re-evaluated by an expert, using modern typochronologies based on C14 dates [29–31]. The locations were also improved using maps (historical and modern) and satellite imagery available through open access web GIS applications. In addition, the data set was enriched with metadata (e.g., the confidence level for chronology and location) and paradata (e.g., sources for dating). Furthermore, it was expanded to include Late Antique sites (400 to 600 CE in our study).

However, this data enrichment focused on a geographically limited subset of data. This subset of 1,106 archaeological sites constitutes the study area of this article. It includes present-day

Slovenia, southern Austria (Carinthia, Styria, East Tyrol, parts of Salzburg and Upper Austria) and a small part of northern Italy (the Trieste region) (Fig. 1).

The data are described in more detail in the data paper [25] and are available via the Zenodo repository [32].

Fig. 1: Map of the study area (upper left corner Lat. 48.22015, Lon. 12.35667; lower right corner Lat. 45.29785, Lon. 16.41784). Study area is marked in red and locations of Fig.s 5a and 5b in black.

Compared to the entirety of Slavic territories ours is a small study region. But this region is an excellent (if not pivotal) case study for understanding the general processes of the spread of Common Slavic for three reasons: (i) it is the only region where Slavic language borders both Germanic and Romance languages; (ii) modern Slovenian (spoken in the southern half of the study region) is the only Slavic language that has so strong links to two groups of Slavic languages (South Slavic and West Slavic), that computing robust NeighborNet network trees is impossible [33]; (iii) the oldest permanent Slavic political entity (*Carantania*, after 650 CE; e.g., [4,22,34–36], the oldest Slavic text other than old church Slavonic (ninth-century *Monumenta Frisingensia*; [37], and the oldest mention of a Slavic elder *župan* (*iopan Physso*, 777 CE; [21,22,38], all originate from this region.

2.2. Space-Time Cube

Artificial Intelligence (AI) is becoming an ever more integral part of the digital humanities. Here, we focus on machine learning, which is a subset of artificial intelligence. It refers to a set of data-driven algorithms and techniques that automate data prediction, classification, and clustering. Machine learning is rapidly being adopted by archaeologists interested in the analysis of

geospatial data, material culture, texts, and artistic data [39,40] and is most often used in combination with Big Data. Among the most prolific fields for machine learning within archaeology is airborne LiDAR data, e.g. overview in [41].

Currently, most machine learning processing takes place in the Phyton and R programming environments. This means that it requires coding and is therefore not readily accessible to most archaeologists. In this paper, we have used one of the few off-the-shelf pipelines that can be readily applied to archaeology: The toolset for space-time pattern mining in ArcGIS Pro v. 2.9 (ESRI, Redlands CA, USA). A similar pipeline has already been demonstrated on archaeological data [42], but to our knowledge ours is the first time this method was used to test a specific archaeological hypothesis.

Some comments on terminology are in order. "Space Time Pattern Mining" is a commercial name for the toolbox used in this work [43]. We classify our method as machine learning, following [40]. They list the methods used in the cultural heritage domain, which are similar to the time series clustering, under unsupervised machine learning. In addition, machine learning is used in the background of this software to enable, for example, intelligent data-driven defaults [44].

Why use space-time pattern mining? Whenever archaeologists (or anyone, for that matter) look at a map, we inherently begin to turn that map into information by finding patterns and assessing trends. However, sometimes the patterns in the data are too complex to be clustered, observed and aggregated by the human eye. In such cases, we can use space-time pattern mining tools to answer our questions confidently, objectively, and repeatably.

The first step in space-time pattern mining is to organize the data. In this case, the data is incorporated into what is known as the "space-time cube model." This model is based on Torsten Hägerstand's time geography, which introduces the time axis into the traditional Cartesian coordinate system, e.g., [45]. It can be thought of as a three-dimensional cube consisting of space-time fields, where the dimensions x and y represent space and the

dimension z represents time (Fig. 2: a). The data is stored in the so-called “space-time NetCDF cube.” The process of constructing the space-time cube data model is complex and beyond the scope of this article, but it is well documented, e.g., [42].

The concept of the space-time cube is familiar to archaeologists. At its core, it is an application of the way we perceive (or have in the past) archaeological excavations: In spatial quadrants and time phases. The quadrants in the xy grid are constant throughout the excavation, and the phases stack on top of each other, with the earliest at the bottom and the latest at the top.

For our case study, we aggregated data into 5 km big (y dimension) and 25 years long (z dimension) hexagonal bins. The size of the hexagon was chosen as the largest in which the relevant processes can be observed; in Early Medieval archaeology, it roughly corresponds to the site catchment area of a single settlement, e.g.,[46]. The time interval of 25 years was determined on the basis of the data properties. The time sensitivity of relevant archaeological dating is about half a century, i.e., ± 25 years. However, start and end dates can often be a quarter of a century, rarely as brief as decades or even years. The accuracy of our dates is therefore 50 years, but the precision is approximately 25 years. Accordingly, 25-year intervals were chosen for analysis, but the accuracy of the data requires that archaeological interpretation be limited to 50-year intervals.

This method is very sensitive to the difference between no data (areas not analyzed) and null data (areas analyzed, but not found to contain any known sites). To account for this, we limited the space-time cube to the area used for data collection. Additionally, we excluded areas higher than 1,400 m above sea level (Fig. 1: shades of brown). In our region, this altitude delineates the highest valley settlements from the lowest high-mountain pastures. The latter were excluded from the analysis because they are specialized seasonal settlements that were always dependent on valley settlements.

Fig. 2. Graphical representations of selected methods: a) space-time cube model (after ESRI); b) comparison of two types of inference: On the left, data from different fields are compiled to draw a unified conclusion (analysis of Pohl's [17] interpretation of our study region as an example), and on the right, consilience.

2.3. Time Series Clustering

Clustering is one of the most widely used machine learning techniques in the field of cultural heritage [40]. Its goal is to organize similar data into homogeneous groups or clusters. Clusters are formed by grouping objects that have maximum similarity with other objects within the cluster and minimum similarity with objects in other clusters. For large and complex data sets, unsupervised approaches offer the best solution. Time series clustering is a type of unsupervised clustering used for data with a temporal component [47,48].

The concept of time- series clustering is deeply familiar to archaeologists having been used since the nineteenth century. At that time, for example, the three-age system, which divides the development of human civilization into the Stone Age, Bronze Age, and Iron Age, was defined by clustering similarly dated stone/bronze/iron artefacts. As most archaeologists know from experience, such clustering is relatively easy for a few artefacts or sites but becomes daunting when the numbers run into the hundreds or thousands of objects or sites. In such cases, unsupervised time series clustering can be used.

In this article, we applied time series clustering to classify sites into chronological groups. In each group, the chronology (start date, end date) of the sites is more similar to each other than that of the sites outside the group.

The similarity between the clusters is measured by the so-called “pseudo-F statistic.” The larger the pseudo-F value, the more different each cluster is from the other clusters [49]. There are several ways to calculate the pseudo-F statistic, each depending on which characteristics of the time series are considered important. In our experience, the most appropriate for archaeology

is the "Profile (Fourier)" method, i.e., method based on Fourier series periodic function. It is used to cluster time series that have similar, smooth, and periodic patterns over time [43].

This method lends itself to the analysis of archaeological processes because they usually follow a consistent pattern: A gradually introduced innovation is followed by a peak of use and a steady decline. Thus, archaeological processes can be compared to seasons, where temperature follows a consistent annual pattern, with higher temperatures in summer and lower temperatures in winter. The “Profile (Fourier)” method is best suited to finding locations that have the most similar annual temperature patterns, for example, to distinguish between locations with mild and severe winters. A season in this example represents an archaeological phase or period.

In our case, we opted to ignore the range, i.e., the magnitude of the values in each period. To extend the analogy above, ignoring the range causes the change of seasons in two places occurring at the same times to be considered similar, even though the actual temperatures are different.

2.4. Modified Emerging Hot Spot Analysis

Spatial analysis is often called upon to determine the density of observed phenomena, and one of the most common tools to do this in archaeology is the so-called “hot spot analysis.” It uses the Getis-Ord G* statistic to calculate z-scores and p-values within a given spatial neighborhood. These indicate whether the observed spatial clustering of high and low values is more (hot spot) or less (cold spot) pronounced than would be expected from a random distribution, e.g., [50].

In this article, we have used an emerging hot spot analysis that examines the clustering of high and low values over time, in addition to spatial trends. The space-time cube is evaluated bin-by-bin, and each bin is analyzed relative to its space-time neighbors. Thus, each site is related not only spatially but also temporally to neighboring sites. The result is similar to the traditional hot spot analysis, except that it is in 3D (where the z dimension represents time).

Such a result can deliver an overwhelming amount of information. Therefore, the tool evaluates the trends of hot spots and cold spots over time (using the Mann-Kendall trend test, e.g., [51] and categorizes each location in the study area accordingly. For example, a location is considered a consecutive hot spot if it has an uninterrupted series of statistically significant hot spot bins over the latest time step intervals, but less than 90% of the total [43]. The resulting 2D representation of trends can be termed a "trend map."

However, the trend map provided by the tool is not suitable for archaeology for two reasons. First, it assumes that the latest records are the focus of analysis. Second, it was designed for data sets much larger than ours and those of most archaeological studies.

We therefore modified the trend map by focusing on chronological periods previously calculated by the time series clustering method. For example, a location was considered a first period consecutive hot spot if it had an uninterrupted hot spot series of at least 100 years within the first period (detailed description in S1 Table). We term this an "archaeological trend map".

In emerging hot spot analysis, the spatial and temporal neighborhoods have a significant influence on the results. We found, through empirical observation, that the best results for hot spots and cold spots were obtained with different settings: (i) fixed distance method with 20 km neighborhood, and time step three for cold spots; (ii) k-nearest neighbors (kNN) method with six spatial neighbors, time step one for hot spots [43].

Therefore, we have introduced another archaeology-specific modification to the tool. For the purposes of this article, we superimposed the cold spots derived using the first calculation (i) with the hot spots derived using the second calculation (ii) in a single visualization. We refer to this method as "multiscale emerging hot spot analysis."

To ensure the highest level of methodological transparency, reproducibility, and transparency and to reduce the time researchers spend replicating the work of other research groups, we provide the ready-to-re-use data in GIS format and the GIS protocol (S2 Appendix).

3. Theory

3.1 Consilience

The specifics of archaeological inference, e.g., [52] are not often outlined in articles such as ours. In this case, however, it is necessary because academic passions on the question of the migration of the Slavs have long been running high, and the methods of inference are often scrutinized. Moreover, this topic is invariably interdisciplinary and, despite (or perhaps because of) its long tradition in archaeology, interdisciplinarity is achieved through a variety of approaches.

In order to enrich this discussion with the most objective archaeological information possible, we have chosen to base our inference on consilience of induction. Consilience, also known as "convergence of evidence," is a scientific principle that states that the same conclusion is much stronger when drawn from independent and unrelated sources. Confidence is strongest when evidence from different fields is considered because the methods and/or data are different [53].

Although it is rarely referred to by its name, this principle is popular in archaeology. For example, consilience is applied whenever radiocarbon dating is invoked to support archaeological dating.

It is important to distinguish between consilience, where conclusions are drawn independently before being correlated, and the more common interdisciplinary approach to the study of Slavic migrations, where data from different fields is compiled to draw a unified conclusion (Fig. 2: b). To this end, we have been careful to consider only information from each field that has not been influenced by findings from another field. For example, in Discussion we consider linguistic information about the verb *gъlčěti, to speak [54], but disregard the conclusions drawn on the same subject matter using supporting evidence from archaeology [55].

3.2 Material Culture as Ethnicity, Identity, and Habitus?

The main archaeological argument for the migrations of the Slavs is based on the association of the Slavs (the people) with various archaeological cultures or *habitus*. For instance, the archaeological assemblages of the so-called Prague Culture are associated with the Early Slavs, e.g., [56–62].

From a modern theoretical perspective, this argument draws on Pierre Bourdieu's [63] notion of *habitus*; its basic premise is that practical knowledge is embodied in daily practices and that material culture, especially pottery, expresses these practices, e.g., [64]. However, equating a *habitus* (e.g., the archaeological assemblages of Prague culture) with a people/tribe/ethnicity (e.g., the Early Slavs) is an additional step that Bourdieu did not anticipate. In much of the literature after the mid-1960s, the notion that material culture is more or less directly related to cognition of peoples was questioned by many; the acceptance that archaeological cultures simply cannot be directly correlated with ethnicity took hold, e.g., [65,66].

Rather than engage in this discourse, we based our argument only on the categories of archaeological data that are indisputable: Location and chronology of the site. Our inference thus eclipses theoretical issues about the associations of material culture with ethnicity, e.g., [67], identity, e.g., [68], or habitus, e.g., [64]. Whether or not the Prague Culture archaeological assemblages are associated with the Early Slavs was immaterial to our conclusions.

4. Results

4.1. Archaeological Periodization

The result of our time series clustering is the archaeological periodization of sites. The most archaeologically meaningful result is when the data is clustered into three periods (pseudo-F statistic value 421,595). These are the Late Antiquity and two Early Middle Ages periods (Fig. 3). The latter two correspond to the traditional periodization of the jewelry into the Carantanian and the Köttlach phases, e.g., [69] or groups A/B and C of Eichert [70].

Fig. 3. Archaeological periodization with time series clustering: LA - Period 1, Late Antiquity; EMA1 - Period 2, Early Middle Ages 1; EMA2 - Period 3, Early Middle Ages 2. Values on x axis are years CE, values on y axis are unitless and relative.

Three important conclusions may be drawn from these results. The first is~~,~~ that the general increase in sites between 400 and 500 CE and the decrease after 1000 CE does not reflect reality, as we know it from numerous archaeological sources, e.g., [22,71]. Rather, it reveals the weakness of the underlying data set: The period before 500 CE has not been collated systematically, and data for the period after 1000 CE (which, until recently, was not considered relevant to archaeology in the region, e.g., [72] is lacking. Regardless, the present data set is suitable for the study of the half millennium between 500 and 1000 CE, which was the aim.

Second, unlike changes in material culture, changes in landscape are more gradual and often overlap. For example, the time series of Late Antiquity does not end until 1000 CE, as some sites exhibit continuity from Late Antiquity onward (for example, the town of Kranj, e.g., [73]. The results of time series analysis in archaeology are therefore complex and must be interpreted with great care.

Fig. 4. Archaeological trend map of the modified categorization of the multiscale emerging hot spot analysis. See S1 Table for the legend.

Third, we substantiated the long-established periodization of the Early Middle Ages into two periods by an independent source of data: The chronology of sites rather than the typology of jewelry. This, then, is the first quantitative evidence that changes in jewelry styles taking place in the second half of the 9th century were reflected in changes in the archaeological landscape.

The most likely explanation is that both changes had the same underlying cause, which however is beyond the scope of this article.

4.2. Archaeological Landscape

The emerging hotspot analysis revealed an astonishing quantity and quality of information (Fig. 4). Most relevant to our topic are the extensive areas of cold spots in the northern part of the region and the general patchiness, i.e. activity is concentrated in enclaves. In this respect, the archaeological landscape between 500 and 1000 CE differs from both the preceding Roman period settlement and subsequent High Medieval period, which both exhibit a more regular pattern of settlement. This is important in providing a context for understanding various historical processes. For example, the reason it is so difficult for historiographers to define the exact borders of *Carniola*, e.g.,[3] and *Carantania*, e.g., [4] is that in the patchy landscape precise fixed borders most likely never existed.

The main focus of this article was migration. The two tools for detecting migrations in our data were provided by Curta [13]. First, migration must have occurred if settlements and cemeteries suddenly appear in a previously sparsely populated area. Second, migration can also be detected by the sudden appearance of a material culture without local traditions or parallels in a given area.

With the first tool, migration was documented in the easternmost part of the study area. It took hold in c. 500 CE along the river Mura (*Ger*. Mur). After a period of consolidation until c. 600 CE, a series of small-scale neighbourhood migrations upstream of the Mura and the adjacent Drava (*Ger*. Drau) rivers ensued into the above mentioned extensive area of cold spots (Fig. 5: a; S3 Appendix).

Fig. 5. Time slices from the emerging hot spot analysis for selected areas: a) Eastern part of the study area from 450 CE to 650 CE; b) central part of the study area from before 600 CE to after 750 CE.

With the second tool, a migration upstream of the Sava river in the late seventh century was documented. During most of the seventh century, there was a decline in archaeological record along the Sava, but in the late seventh and early eighth centuries there was a reversal of that trend (Fig. 5: b; S3 Appendix). However, this reversal on its own could be explained by causes other than migration. The evidence for migration was provided by the time series clustering, which showed a sudden and complete shift in material culture, i.e. sites in the landscape (Fig. 3). This shift has long been known in archaeology as the transition from fortified hilltop settlements to unfortified lowland settlements, e.g., [71].

On the basis of this data, a comment can be made on the size of the migrations. Overall, hot spots interpreted as resulting from the first migration account for only 4 % of all hot spots in c. 500 CE, and by c. 700 CE, 59 % of hot spots can be interpreted as resulting directly or indirectly from both migrations. Although this is a very rough estimate, far from giving a direct indication of the actual number of people involved, it is the best available and by far the most tangible to date, *cf.*, [3,4,38,74–77]. As such, it is an invaluable foundation for explaining acculturation processes following migrations, which, however, are not the subject of this article.

5. Discussion

5.1 Archaeology

Our data thus testifies to two migrations: The first upstream of the Mura and Drava rivers after c. 500 CE, and the second before c. 700 CE upstream of the Sava river. This is an important discovery, but it does not shed light on who the migrants were. Based on the archaeological [78–81]) and historiographical [17,20,22] context, we can hypothesize that they were Early

Slavs. But this hypothesis inherits all the weaknesses of existing hypotheses based on the presence of archaeological assemblages of Prague Culture and scant written sources.

The hypothesis may, however, be considered the null hypothesis that can be tested with the consilience principle. The new archaeological evidence for two separate migrations allows us to correlate it with information from the linguistics and genetics of modern populations. These two fields of science use completely different data sources and methods than archaeology and have recently made significant advances in understanding Slavic migrations.

5.2 Linguistics

Let us first take a look at linguistics. Modern linguistics holds that, while a language or dialect may be tied to any number of identities within a given period, a common linguistic innovation requires the community in which it occurs, the so-called "founder population" [6].

Based on this premise, the vocabulary of modern Slovenian - spoken today in the southern part of the region under study - shows a considerable degree of mixture with West and South Slavic languages, i.e., Slovenian belongs to the South Slavic clade but has considerable linguistic similarities with West Slavic lects, e.g., [82,83]. The latter has recently been corroborated with NeighborNet phylogenetic networks of Balto-Slavic languages [33].

Looking more closely at Slovenian dialects, there is evidence that the Slovenian territory was originally populated by speakers of heterogeneous Slavic dialects. In particular, lexical evidence points to a distinct founder population in the area along the Mura and Drava rivers, which likely predated other Slavic immigrant groups. This is evidenced by the parallel development of the Slavic inherited vocabulary *gъlčěti- (to speak), which is found only in distal locations along the Mura and Drava Rivers, in Bulgaria, and in central European Russia [54].

Therefore, linguistic studies show that the southern part of the region under study was probably settled in two separate migrations. The earlier one ran along the Mura and Drava rivers, the later one cannot yet be geographically determined by linguistics.

5.3 Genetic history

Second, let us turn to genetic history. This scientific field attempts to reconstruct human evolution and the history of human populations using genetic information obtained from either modern or ancient DNA [84]. DNA has been described as a document containing "messages from the past" [85] and is a proven tool in prehistoric archaeology, e.g., [86–89]. However, there are significant obstacles to the use of modern DNA when it comes to Late Antiquity and the Middle Ages [90,91]. For this time period, ancient DNA or ancient genomic DNA data are more appropriate, e.g., [92,93]. However, ancient DNA data are not available in sufficient quantity to study the expansion of Slavic languages.

Regardless of the methodological shortcomings, it is agreed that the results of genetic studies on modern DNA are indisputable in terms of providing information on genetic proximity and can contribute to hypotheses about human population history, including Late Antiquity and Early Mediaeval migrations [90,93–95].

The most complete study of modern DNA pertaining to the expansion of Slavic languages examined all ethnic groups living today who speak Balto-Slavic languages, as well as their neighbors. It concluded that the genetic diversity of today's Slavs was predominantly formed *in situ* (i.e., the substrate genetic components in the settled areas prevail), with marked differences between West and East Slavs on the one hand and South Slavs on the other. However, there is genetic affinity showing a common ancestry (i.e., a homogeneous genetic substrate inherited from the ancestral population) among the Slavs, which probably demonstrates the historical dispersion of the once uniform population [33]. This was recently confirmed in a review article,

which concluded, that the migration of Slavs was accompanied by active assimilation of indigenous European populations [96].

Looking more closely at the region under study, the variability of the microsatellite loci of the Y chromosome is telling. It shows that Slovenians and West Croats are far removed from all other South Slavic populations [33]. This could have been caused by at least two different migrations of Slavs in the Balkans from different regions of Europe [97].

Considering only the modern Slovenian population, there is one possible ancestral haplogroup for all Slovenian populations which has the highest frequency along the Mura and Drava rivers. This could indicate that the origin of this ancestral haplogroup was in this area and that it later spread westward [98].

Thus, population genetic studies show that the southern part of the study region was probably settled by two separate migrations. The earlier one took place along the Mura and Drava rivers, and the later one, which cannot be geographically determined by genetics.

5.4 Consilience

Information from three scientific fields, using completely different data sets and methods, shows a clear consilience or convergence of evidence. Archaeology, linguistics, and population genetics have each concluded that there were two separate migrations to the southern part of the region under study: The earlier one along the Mura and Drava rivers, and the later one, which only archaeology can locate along the Sava river. Archaeology and genetics have concluded that acculturation was the predominant post-migration process. Linguistics and genetics have concluded that the migrants were Slavs. In particular, linguistics confirms that the migrants were speakers of Common Slavic, and genetics confirms that they had a homogeneous genetic substrate inherited from a single ancestral population common to all ethnic groups speaking Slavic languages alive today (Table 1).

Table 1. Consilience or Convergence of Evidence (- is a null hypothesis).*

	Archaeology	Linguistics	Genetics
Two separate migrations (earlier on Mura and Drava r.)	YES	YES	YES
Strong acculturation	YES	NO	YES
Migrants are Slavs	YES*	YES	YES

On this basis, we can define the immigrants who arrived in the Eastern Alps between c. 500 and c. 700 CE with by far the highest reliability to date. They were speakers of Common Slavic and shared specific ancestry, which was in common to them and is in common to modern speakers of Slavic language (but was not, for example, in common with their neighbors). Our archaeological analysis places these migrations in space and time with some precision (Fig. 5).

6. Conclusions

The aim of this article was to test the hybrid hypothesis of Slavic migration using archaeological data, with the Alpine Slavs as a case study. The term "migration" in the title was deliberately chosen to be somewhat provocative, as modern historiography and archaeology of the Early Middle Ages tend to downplay the role of physical migrations.

We used selected machine learning methods to analyze an archaeological data set that can be described as Deep Data. Specifically, we used two methods: Time series clustering and a modified emerging hot spot analysis. The former method is directly suitable for archaeology without modification, whereas the latter required two archaeology-specific modifications: The archaeological trend map and the multiscale emerging hot spot analysis.

The results have provided us with an overwhelming quality and quantity of new information. In this article, we have focused on confirming two separate migrations of the Alpine Slavs. Based on the convergence of evidence from archaeology, linguistics, and population genetics, we define the immigrants as Alpine Slavs who were speakers of Common Slavic and shared specific ancestry, which was in common to them and is in common to modern speakers of Slavic language. They migrated to the Eastern Alps on two occasions, first after c. 500 and second before c. 700 CE.

The identities and ethnicity of the migrants are, in our view, beyond the scope of archaeology. The acculturation processes that took place after the migration will be discussed elsewhere. From the available evidence, however, it is clear that the crucial process was cultural spread *sensu* Heather [18]. We envisage that the number of migrating people was relatively small and more akin to a small group infiltration than a mass migration. The movement itself was a part of it, but the processes that took place afterwards were historically the most important.

Thus, we have achieved the aim of the article, which was to prove the validity of the hybrid hypothesis of Slavic migration with archaeological data. The migration of the Alpine Slavs was a combination of movement of people, cultural diffusion, and language diffusion, all occurring simultaneously. This clearly refutes the hypothesis that only the cultural model or even only the language spread.

While this paper focused on a specific question related to the migration of Slavs, the methods we developed, borrowing and adapting from a variety of disciplines, can be applied to archaeological studies of any period, anywhere that sufficient data is available. We hope that these advances will be used beneficially by other scholars and establish a new, practical approach to add to the archaeological arsenal of methodologies.

In the field of machine learning in archaeology and the digital humanities in general, we hope to have shown that, in addition to Big Data, Deep Data also holds great potential.

7. Acknowledgments

Author Contributions

Benjamin Štular: conceptualization, methodology, software, writing—original draft preparation, writing—review and editing, supervision, project administration, funding acquisition.

Edisa Lozić: Conceptualization, methodology, validation, formal analysis, writing—original draft preparation, writing—review and editing, visualization.

Mateja Belak: investigation, data curation.

Jernej Rihter: investigation.

Iris Koch: investigation.

Zvezdana Modrijan: investigation.

Andrej Magdič: investigation, writing—review and editing, project administration.

Stephan Karl: investigation, writing—review and editing, project administration.

Manfred Lehner: writing—review and editing, supervision, project administration.

Christoph Gutjahr: project administration.

All authors have read and agreed to the published version of the manuscript.

Funding: This research was funded by Austrian Science Fund (FWF) grant number I 3992, and Slovenian Research Agency (ARRS) grant numbers J6-9450 and P6-0064.

Data Availability Statement

Data used in this article are available under a CC-By licence at https://doi.org/10.5281/zenodo.5761811 and https://zenodo.org/record/5813527.

Acknowledgments: The authors give thanks for the constructive comments and suggestions by academic editors and the anonymous reviewers.

Conflicts of Interest: The authors declare no conflict of interest. The funders had no role in the design of the study, in the collection, analyses, or interpretation of data, in the writing of the manuscript, or in the decision to publish the results.

8. References

1. Deacon P, Foulke WD. History of the Lombards. Peters E, editor. Philadelphia: University of Pennsylvania Press; 1974.

2. Bradač F, Grafenauer B, Gantar K. Pavel Diakon (Paulus Diaconus), Zgodovina Langobardov (Historia Langobardorum). Maribor: Obzorja; 1988.

3. Bratož R. Med Italijo in Ilirikom. Slovenski prostor in njegovo sosedstvo v pozni antiki. Ljubljana: Filozofska fakulteta; 2014. (Zbirka ZČ 46).

4. Kahl H-D. Der Staat der Karantanen. Fakten, Thesen und Fragen zu einer frühen slawischen Machtbildung im Ostalpenraum (7.-9. Jh.). Bratož R, editor. Ljubljana: Narodni muzej Slovenije; 2002.

5. Pohl W. The Early Slavs: Culture and Society in Early Medieval Eastern Europe. P. M. Barford. Speculum. 2004;79: 448–450. doi:10.1017/s0038713400087996.

6. Snoj M, Greenberg ML. O jeziku slovanskih prebivalcev med Donavo in Jadranom v srednjem veku (pogled jezikoslovcev). Zgodovinski časopis. 2012;66: 276–305.

7. Curta F. Slavs in the Making: History, Linguistics, and Archaeology in Eastern Europe (ca. 500-ca. 700). London, New York: Routledge; 2020. doi:10.4324/9780203701256.

8. Herrmann J. Urheimat und Herkunft der Slawen. In: Herrmann J, editor. Welt der Slawen. Geschichte, Gesellschaft, Kultur. München: Verlag C. H. Beck; 1986. pp. 11–18.

9. Dolukhanov P. The early Slavs: Eastern Europe from the initial settlement to the Kievan Rus. London, New York: Routledge; 1996.

10. Timberlake A. Culture and the spread of Slavic. In: Bickel B, Grenoble LA, Peterson DA, Timberlake A, editors. Language Typology and Historical Contingency: In honor of Johanna Nichols. Amsterdam, Ph: John Benjamin's Publishing Company; 2013. pp. 331–356.

11. Lunt HG. Common Slavic, Proto-Slavic, Pan-Slavic: What Are We Talking About? International Journal of Slavic Linguistics and Poetics. 1997;41: 7–67.

12. Curta F. The Making of the Slavs : History and Archaeology of the Lower Danube Region, c. 500-700. Cambridge: Cambridge University Press; 2001.

13. Curta F. Migrations in the Archaeology of Eastern and Southeastern Europe in the Early Middle Ages (Some Comments on the Current State of Research). In: Preise-Kapeller J, Reinfandt L, Stouraitis Y, editors. Migration Histories of the Medieval Afro-Eurasian Transition Zone: Aspects of mobility between Africa, Asia and Europe, 300-1500 CE. London, New York: Brill; 2020. pp. 101–140.

14. Pritsak O. The Slavs and the Avars. In: Gli slavi occidentali e meridionali nell'alto medioevo 15 - 21 Aprile 1982 Settimane di Studio del Centro Italiano di Studi Sull'Alto Medioevo, XXX. Fondazione Centro italiano di studi sull'alto Medioevo; 1983. pp. 353–435.

15. Kazanski M. Archaeology of the Slavic Migrations. In: Greenberg ML, Grenoble LA, editors. Encyclopedia of Slavic Languages and Linguistics. London, New York: Brill; 2020. doi: 10.1163/2589-6229_ESLO_COM_035967.

16. Pleterski A. Etnogeneza slavena - metode i proces. Starohrvatska prosvjeta. 2013;40: 8–32.

17. Pohl W. The Avars: a Steppe Empire in Europe, 567-822. Ithaca, NY: Cornell University Press; 2018. doi: 10.7591/9781501729409.

18. Heather P. Empires and barbarians: The fall of Rome and the birth of Europe. Oxford: Oxford University Press; 2010.

19. Lindstedt J, Salmela E. Language Contact and the Early Slavs. In: Klír T, Boček V, Jansens N, editors. New perspectives on the Early Slavs and the rise of Slavic. Universitätsverlag Winter GmbH; 2020. pp. 275–299.

20. Grafenauer B. Zgodovina Slovenskega naroda, I. zvezek: Od naselitve do uveljavljenja frankovskega fevdalnega reda. Ljubljana: Kmečka knjiga; 1954.

21. Friesinger H. Alpenslawen und Bayern. In: Herrmann J, editor. Welt der Slawen. Geschichte, Gesellschaft, Kultur. München: Verlag C. H. Beck; 1986. pp. 109–122.

22. Štih P. The Middle Ages between the Eastern Alps and the Northern Adriatic. Select Papers on Slovene Historiography and Medieval History. London, New York: Brill; 2010.

23. Pleterski A. Zbiva v3.08; 2016. [cited 2021 Dec 30]. Database: Sites and Monuments [Internet]. Available from: http://zbiva.zrc-sazu.si

24. Štular B. The Zbiva Web Application: a tool for Early Medieval archaeology of the Eastern Alps. In: Richards JD, Niccolucci F, editors. The ARIADNE Impact. Budapest: Archaeolingua; 2019. pp. 69–82. doi: 10.5281/zenodo.3476712.

25. Štular B, Belak M. Making Deep Data FAIRrer. Zbiva, Early Medieval Data Set for the Eastern Alps. Research Data Journal for the Humanities and Social Sciences. 2022;Submitted for publication.

26. Huggett J. Is Less More? Slow Data and Datafication in Archaeology. In: Garstki K, editor. Critical Archaeology in the Digital Age. Los Angeles, CA: Cotsen Insititute of Archaeology; 2022. pp. 156–184.

27. Filzwieser R, Eichert S. Towards an Online Database for Archaeological Landscapes. Using the Web Based, Open Source Software OpenAtlas for the Acquisition, Analysis and Dissemination of Archaeological and Historical Data on a Landscape Basis. Heritage. 2020;3: 1385–1401. doi:10.3390/heritage3040077.

28. Eichert S. Digital Mapping of Medieval Cemeteries. Journal on Computing and Cultural Heritage. 2021;14: 1–15. doi: 10.1145/3406535.

29. Pleterski A. Datiranje zgodnjesrednjeveške naselbine Lehen pri Mitterkirchnu v Zgornji Avstriji kot kontrola nove datacijske metode s pomočjo referenčne tabele in korelacijske formule ustij loncev. Vjesnik Arheološkog muzeja u Zagrebu. 2010;43: 309–324.

30. Pleterski A. Zgodnjesrednjeveška naselbina na blejski Pristavi : tafonomija, predmeti in čas. Ljubljana: Založba ZRC; 2010.

31. Pleterski A. A step towards the chronology of early medieval head ornaments in the Eastern Alps. Arheološki vestnik. 2013;64: 299–334.

32. Štular B, Pleterski A, Belak M. Zbiva, Early Medieval Data Set for the Eastern Alps. Data sub-set. 2021. [cited 2021 Dec 29]. Repository: Data Set [Internet]. Available from: doi:10.5281/ZENODO.5761811

33. Kushniarevich A, Utevska O, Chuhryaeva M, Agdzhoyan A, Dibirova K, Uktveryte I, et al. Genetic Heritage of the Balto-Slavic Speaking Populations: A Synthesis of Autosomal, Mitochondrial and Y-Chromosomal Data. Calafell F, editor. PLOS ONE. 2015;10: e0135820. doi:10.1371/journal.pone.0135820

34. Gleirscher P. Karantanien: slawisches Fürstentum und bairische Grafschaft. Klagenfurt/Celovec: Hermagoras Verlag., Mohorjeva založba; 2018.

35. Eichert S. Karantanische Slawen - slawische Karantanen. Überlegungen zu ethnischen und sozialen Strukturen im Ostalpenraum des frühen Mittelalters. In: Biermann F, Kersting, T, Klammt, A. Der Wandel um 1000 Beiträge der Sektion zur slawischen Frühgeschichte der 18. Jahrestagung des Mittel- und Ostdeutschen Verbandes für Altertumsforschung in Greifswald, 23. bis 27. März 2009. Langenweissbach: Beier & Beran; 2011. pp. 433–440.

36. Pleterski A. Die Kärntner Fürstensteine in der Struktur dreier Kultstätten. In: Huber A, editor. Der Kärntner Fürstenstein im europäischen Vergleich (Symposium Gmünd 1996). Gmünd: Die Stadt; 1997. pp. 43–119.

37. Ogrin M, Grdina I, Erjavec T, Bojadžiev D. Brižinski spomeniki: Monumenta Frisingensia: elektronska znanstvenokritična izdaja. Ljubljana: Inštitut za slovensko literaturo in literarne vede ZRC SAZU; 2007. Available from: http://nl.ijs.si/e-zrc/bs/html/bs.html.

38. Štih P. Strukture današnjega slovenskega prostora v zgodnjem srednjem veku. In: Bratož R, editor. Slowenien und die Nachbarländer zwischen Antike und karolingischer Epoche. Anfänge der slowenischen Ethnogenese I. Ljubljana: Narodni muzej Slovenije; 2000. pp. 355-394.

39. Bickler SH. Machine Learning Arrives in Archaeology. Advances in Archaeological Practice. 2021;9: 186–191. doi:10.1017/aap.2021.6.

40. Fiorucci M, Khoroshiltseva M, Pontil M, Traviglia A, Del Bue A, James S. Machine Learning for Cultural Heritage: A Survey. Pattern Recognition Letters. 2020;133: 102-108. doi: 10.1016/j.patrec.2020.02.017.

41. Lozić E, Štular B. Documentation of Archaeology-Specific Workflow for Airborne LiDAR Data Processing. Geosciences. 2021;11: 26. doi:10.3390/geosciences11010026

42. Cui J, Liu Y, Sun J, Hu D, He H. G-STC-M Spatio-Temporal Analysis Method for Archaeological Sites. ISPRS International Journal of Geo-Information. 2021;10: 312. doi: 10.3390/ijgi10050312.

43. ESRI. How Create Space Time Cube works. 2021. [cited 29 December 2021]. In: ESRI Help [Internet]. Available from: https://pro.arcgis.com/en/pro-app/latest/tool-reference/space-time-pattern-mining/learnmorecreatecube.htm.

44. Bennett L. Machine Learning in ArcGIS. ArcUser, the Magazine for Esri Software Users . 2018;21(2): 8-9. Available from: https://www.esri.com/about/newsroom/arcuser/machine-learning-in-arcgis/.

45. Kraak M-J. Geovisualization and time: new opportunities for the space-time cube. In: Dodge M, McDerby M, Turner M, editors. Geographic visualization: concepts, tools and applications. Chichester, England; Hoboken, NJ: John Wiley & Sons, Ltd; 2008. pp. 293–306. doi:10.1002/9780470987643.ch15

46. Lozić E. Application of Airborne LiDAR Data to the Archaeology of Agrarian Land Use: The Case Study of the Early Medieval Microregion of Bled (Slovenia). Remote Sensing. 2021;13(16): 3228. doi:10.3390/rs13163228.

47. Montero P, Vilar JA. TSclust: An R package for time series clustering. Journal of Statistical Software. 2014;62: 1–43. doi: 10.18637/jss.v062.i01.

48. Aghabozorgi S, Shirkhorshidi AS, Wah TY. Time-series clustering - A decade review. Information Systems. 2015;53: 16–38. doi:10.1016/j.is.2015.04.007.

49. Vogel MA, Wong AKC. PFS clustering method. IEEE transactions on pattern analysis and machine intelligence. 1979; 237–245.

50. Achino KF, Štular B, Rihter J, Rihter J. Assessing the intentionality of spatial organization. Cemetery of Župna Cerkev (Kranj, Slovenia) case study. Arheološki vestnik. 2019;70: 297–313.

51. Hamed KH, Rao AR. A modified Mann-Kendall trend test for autocorrelated data. Journal of hydrology. 1998;204: 182–196.

52. Hodder I. The Archaeological Process an Introduction. Oxford: Wiley-Blackwell; 1999.

53. Wilson EO. Consilience. The unity of knowledge. New York: Vintage Books; 1998.

54. Schallert J, Greenberg ML. The Prehistory and Areal Distribution of Slavic *gъlčěti `Speak’. Slovenski jezik / Slovene Linguistic Studies. 2007;6: 9–76.

55. Greenberg ML. Slavs as migrants: mapping prehistoric language variation. In: Genis R, de Haard E, Lučić R, editors. Definitely Perfect Festschrift for Janneke Kalsbeek. Amsterdam: Uitgeverij Pegasus; 2017. pp. 169–183.

56. Kazanski M. Les Slaves. Les origines (Ier-VIIe siècle après J.-C.). Paris: Editions errance; 1999.

57. Barford PM. The early Slavs: culture and society in early medieval Eastern Europe. Ithaca: Cornell University Press; 2001.

58. Gojda M. The Ancient Slavs; Settlement and Society. Edinburgh: Edinburgh University Press; 1991.

59. Parczewski M. Origins of early Slav culture in Poland. Antiquity. 1991;65: 676–683. doi: 10.1017/S0003598X00080303.

60. Biermann, F, 2016 New archaeological evidence from the Late Migration and Early Slavic period in the north-east German region. In: Chudzin´ska, B, Wojenka, M, Wołoszyn, M, editors. Od Bachórza Do Swiatowida Ze Zbrucza: Tworzenie Się Słowianskiej Europy W Ujęciu Zródłoznawczym: Księga Jubileuszowa Profesor a Michała Parczewskiego. Kraków-Rzeszów: Wydawnictwo Uniwersytetu Rzeszowskiego; 2016. pp. 113–123.

61. Pavlovič D, Vojakovič P, Toškan B. Cerklje ob Krki: novosti v poselitvi Dolenjske v zgodnjem srednjem veku. Arheološki vestnik. 2021;72: 137–186. doi:10.3986/av.72.06

62. Macháček J, Nedoma R, Dresler P, Schulz I, Lagonik E, Johnson SM, et al. Runes from Lány (Czech Republic) - The oldest inscription among Slavs. A new standard for multidisciplinary analysis of runic bones. Journal of Archaeological Science. 2021;127: 105333. doi: 10.1016/j.jas.2021.105333.

63. Bourdieu P. Outline of a Theory of Practice. Cambridge: Cambridge University Press; 1977.

64. Skibo JM, Schiffer MB. People and Things. A Behavioral Approach to Material Culture. New York: Springer; 2008.

65. Jones S. The Archaeology of Ethnicity: A Theoretical Perspective. London, New York: Routledge; 1997.

66. Brather S. Ethnische Identitäten als Konstrukte der frühgeschichtlichen Archäologie. Germania: Anzeiger der Römisch-Germanischen Kommission des Deutschen Archäologischen Instituts. 2000;78: 139–177.

67. Hu D. Approaches to the Archaeology of Ethnogenesis: Past and Emergent Perspectives. Journal of Archaeological Research. 2012; 371–402.

68. Dzino D. Becoming Slav, Becoming Croat. Identity Transformations in Post-Roman and Early Medieval Dalmatia. Leiden, Boston: Brill; 2010.

69. Korošec P. Zgodnjesrednjeveška arheološka slika karantanskih Slovanov. Ljubljana: SAZU; 1979.

70. Eichert S. Die frühmittelalterlichen Grabfunde Kärntens: Die materielle Kultur Karantaniens anhand der Grabfunde vom Ende der Spätantike bis ins 11. Jahrhundert. Klagenfurt: Verlag des Geschichtsvereines für Kärnten; 2010.

71. Ciglenečki S. Archaeological investigations of the decline of antiquity in Slovenia. In: Bratož R, editor. Slowenien und die Nachbarländer zwischen Antike und karolingischer Epoche. Anfänge der slowenischen Ethnogenese I. Ljubljana: Narodni muzej Slovenije; 2000. pp. 119–139.

72. Predovnik KK, Nabergoj T. Archaeological research into the periods following the Early Middle Ages in Slovenia. Arheološki vestni. 2010;61: 245–294.

73. Kosi M. Predurbane ali zgodnjeurbane naselbine? : (Civitas Petouia, Carnium/Creina in druga centralna naselja neagrarnega značaja v zgodnjem srednjem veku). Del 2. Zgodovinski časopis. 2010;64: 8–44.

74. Grafenauer B. Ustoličevanje koroških vojvod in država karantanskih Slovencev. Ljubljana: SAZU; 1952.

75. Grafenauer B. Zgodovina Slovenskega naroda, I. zvezek: Od naselitve do uveljavljenja frankovskega fevdalnega reda. Ljubljana: Državna založba Slovenije; 1978.

76. Vilfan S. Zur Struktur der freisingischen Herrschaften südlich der Tauern in Frühmittelalter. In: Hödl G, Grabmayer J, editors. Karantanien und der Alpen-Adria-Raum im Frühmittelalter 2 St Veiter Historikergespräche. Wien: Böhlau; 1993. pp. 209–222.

77. Szameit E. Zum archäologischen Bild der frühen Slawen in Österreich. Mit Fragen zur ethnischen Bestimmung karolingerzeitlicher Gräberfelder im Ostalpenraum. In: Bratož R, editor. Slowenien und die Nachbarländer zwischen Antike und karolingischer Epoche. Anfänge der slowenischen Ethnogenese I. Ljubljana: Narodni muzej Slovenije; 2000. pp. 507–548.

78. Pavlovič D. Začetki zgodnjeslovanske poselitve Prekmurja = Beginnings of the Early Slavic settlement in the Prekmurje region, Slovenia. Arheološki vestnik. 2017;68: 349–386.

79. Guštin M, Tifengraber G.. Oblike in kronologija zgodnjesrednjeveške lončenine na Novi tabli pri Murski Soboti = Formen und Chronologie frühmittelalterlicher Keramik in Nova tabla bei Murska Sobota. In: Guštin M, editor. Zgodnji Slovani. Zgodnjesrednjeveška lončenina na obrobju vzhodnih Alp (Die frühen Slawen. Frühmittelalterliche Keramik am Rand der Ostalpen). Narodni Muzej Slovenije; 2002. pp. 46–64.

80. Novšak M. Zgodnjesrednjeveške najdbe z najdišča Grofovsko pri Murski Soboti. In: Guštin M, editor. Zgodnji Slovani. Zgodnjesrednjeveška lončenina na obrobju vzhodnih Alp (Die

frühen Slawen. Frühmittelalterliche Keramik am Rand der Ostalpen). Narodni Muzej Slovenije; 2002. pp. 27–32.

81. Kerman B. Arheološka slika slovanske poselitve Prekmurja = Archaeological picture of the settlement of the Slavs in Prekmurje. In: Lux J, Štular B, Zanier K, editors. Slovani, naša dediščina = Our heritage: the Slavs. Ljubljana: Zavod za varstvo kulturne dediščine Slovenije; 2018. pp. 55–68.

82. Sobolev AN. Voprosy drevnejshej istorii yuzhnoslavyanskikh yazykov i areal'naya lingvistika. Južnoslovenski filolog. 2000;56: 1035–1050.

83. Bezlaj F. Položaj slovenščine v okviru slovanskih jezikov. In: Furlan M, editor. Zbrani jeziskoslovni spisi. Ljubljana: Založba ZRC; 2003. pp. 268–277.

84. Wawruschka C. Genetic History and Identity: The Case of Turkey. Medieval Worlds. 2016;4: 123–161. doi:10.1553/medievalworlds_no4_2016s123.

85. Sykes B. The Seven Daughters of Eve and Blood of the Isles: Exploring the Genetic Roots of our Tribal History. London: Transworld Corgi; 2007.

86. Mathieson I, Lazaridis I, Rohland N, Mallick S, Patterson N, Roodenberg SA, et al. Genome-wide patterns of selection in 230 ancient Eurasians. Nature. 2015;528(7583): 499–503. doi:10.1038/nature16152.

87. Lazaridis I, Nadel D, Rollefson G, Merrett DC, Rohland N, Mallick S, et al. Genomic insights into the origin of farming in the ancient Near East. Nature. 2016;536(7617): 419–424. doi:10.1038/nature19310.

88. Lipson M, Cheronet O, Mallick S, Rohland N, Oxenham M, Pietrusewsky M, et al. Ancient genomes document multiple waves of migration in Southeast Asian prehistory. Science. 2018;361: 92–95. doi:10.1126/science.aat3188.

89. Gokcumen O, Frachetti M. The Impact of Ancient Genome Studies in Archaeology. Annual Review of Anthropology. 2020;49: 277–298. doi:10.1146/annurev-anthro-010220-074353.

90. Geary P, Veeramah K. Mapping European Population Movement through Genomic Research. Medieval Worlds. 2016;4:: 65–78. doi:10.1553/medievalworlds_no4_2016s65.

91. Brather S, editor. New Questions Instead of Old Answers: Archaeological Expectations of aDNA Analysis. Medieval Worlds. 2016;4: 22–41. doi:10.1553/medievalworlds_no4_2016s22.

92. O'Sullivan N, Posth C, Coia V, Schuenemann VJ, Price TD, Wahl J, et al. Ancient genome-wide analyses infer kinship structure in an Early Medieval Alemannic graveyard. Science Advances. 2018;4(9): eaao1262. doi:10.1126/sciadv.aao1262.

93. Amorim CEG, Vai S, Posth C, Modi A, Koncz I, Hakenbeck S, et al. Understanding 6th-century barbarian social organization and migration through paleogenomics. Nature Communications. 2018;9. doi:10.1038/s41467-018-06024-4

94. Pohl W. Editor's Introduction: The Genetic Challenge to Medieval History and Archaeology. P Medieval Worlds. 2016;4: 2–4. doi:10.1553/medievalworlds_no4_2016s2.

95. Samida S, Feuchter J. Why Archaeologists, Historians and Geneticists Should Work Together – and How. Medieval Worlds. 2016;4: 5–21. doi:10.1553/medievalworlds_no4_2016s5.

96. Malyarchuk BA, Derenko MV. Diversity and Structure of Mitochondrial Gene Pools of Slavs in the Ethnogenetic Aspect. Biology Bulletin Reviews. 2021;11: 122–133. doi: 10.1134/s2079086421020067.

97. Malyarchuk BA, Grzybowski T, Derenko MV, Czarny J, Drobnič K, Miścicka-Śliwka D. Mitochondrial DNA variability in Bosnians and Slovenians. Annals of human genetics. 2003;67: 412–425. doi: 10.1046/j.1469-1809.2003.00042.x.

98. Zupan A. Genetska struktura Slovencev, kot jo razkrivajo polimorfizmi kromosoma Y in mitohondrijske DNA. Doctoral Thesis, The University of Ljubljana. 2014. Available at: https://repozitorij.uni-lj.si/IzpisGradiva.php?id=76713.

9. Supporting information captions

S1 Table. Archaeological trend map, detailed description of 16 classes.

S2 Appendix. GIS Protocol for multy-scale emerging hot spot analysis.

S3 Appendix. Animation of the emerging hot spot analysis time slices from 400 CE to 1100 CE.

Figure
Click here to access/download;Figure;StularEt_Figure1.pdf

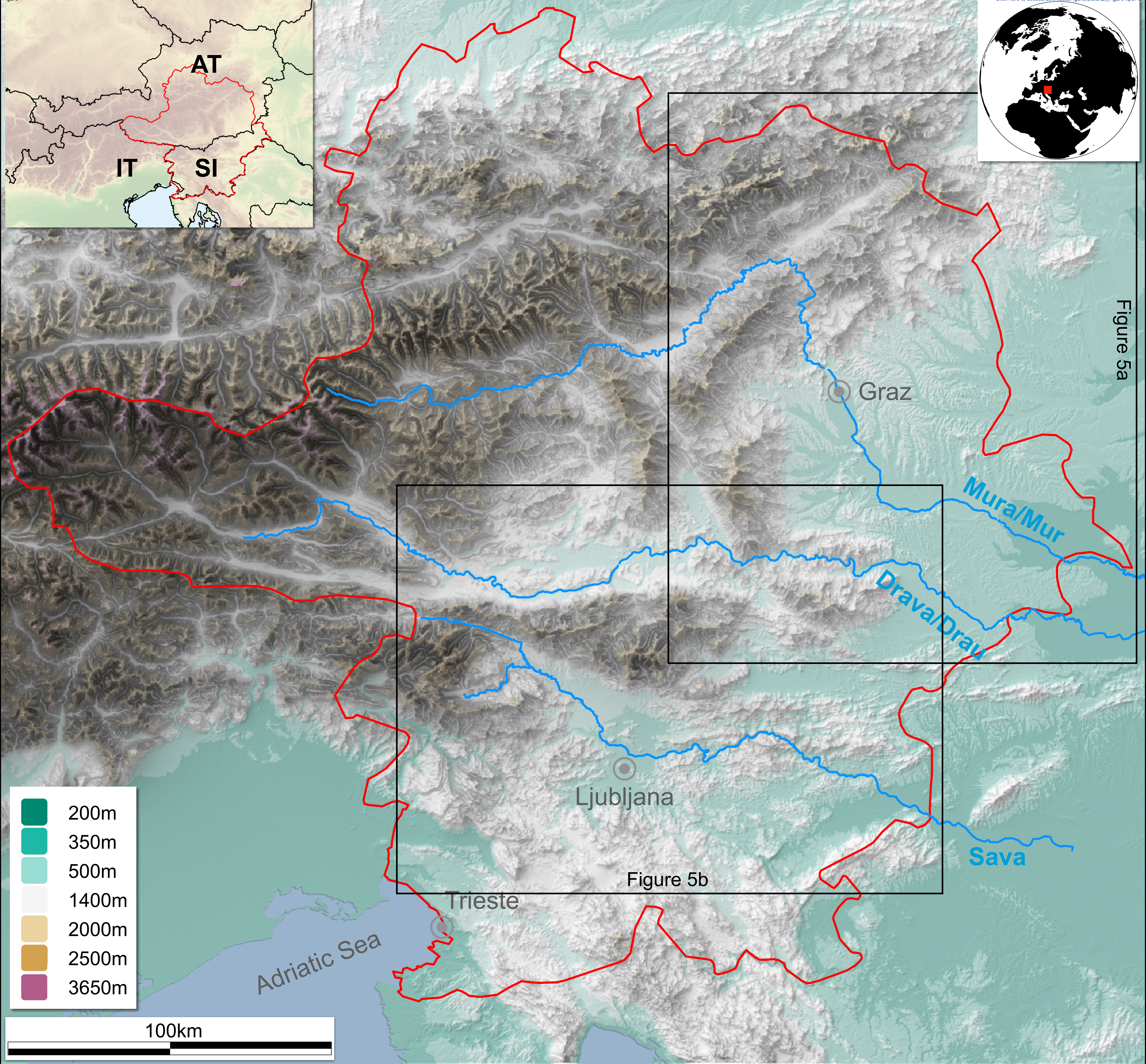

 Click here to access/download;Figure;StularEt_Figure2.pdf

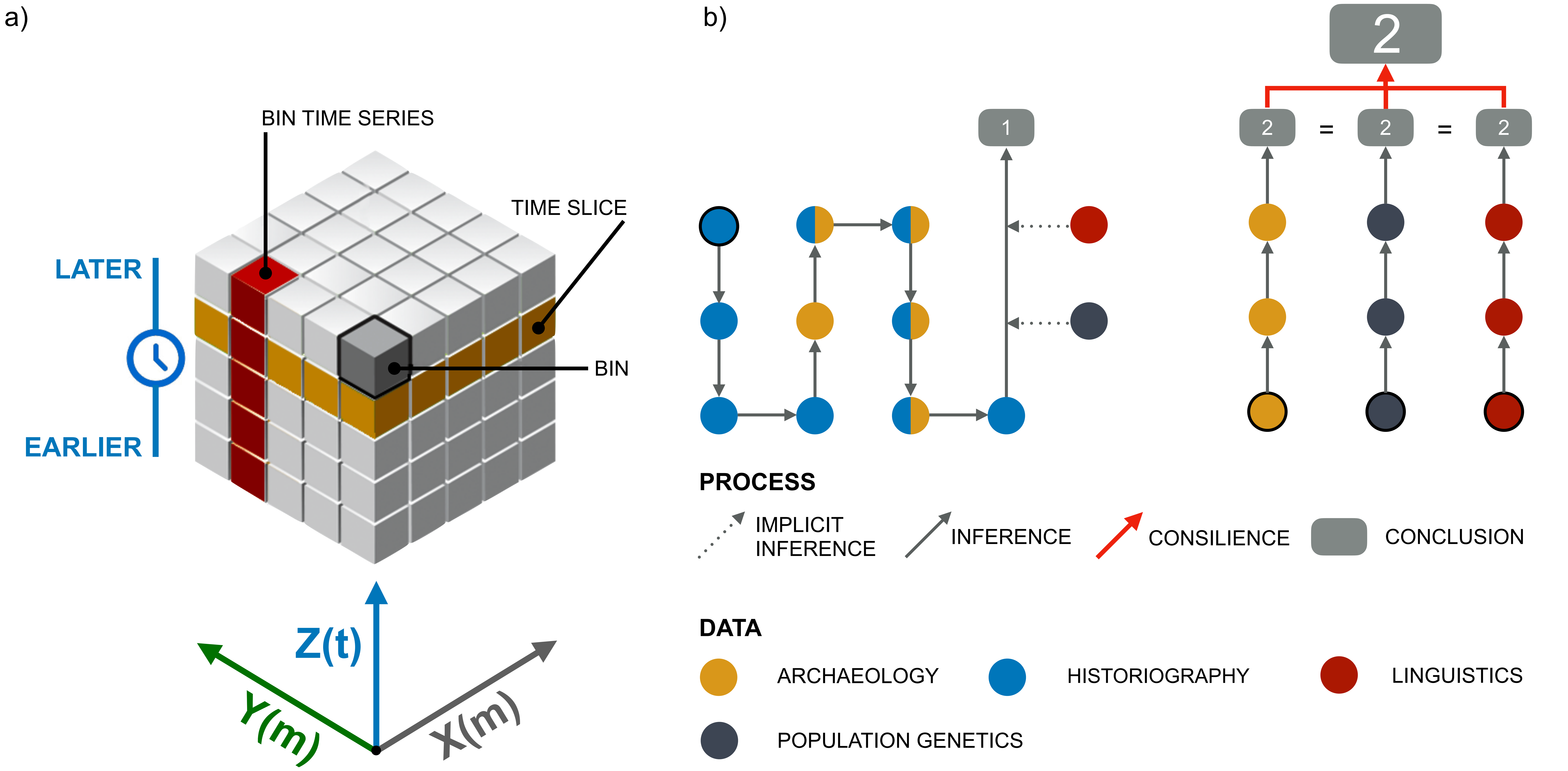

Figure

Click here to access/download;Figure;StularEt_Figure3.pdf

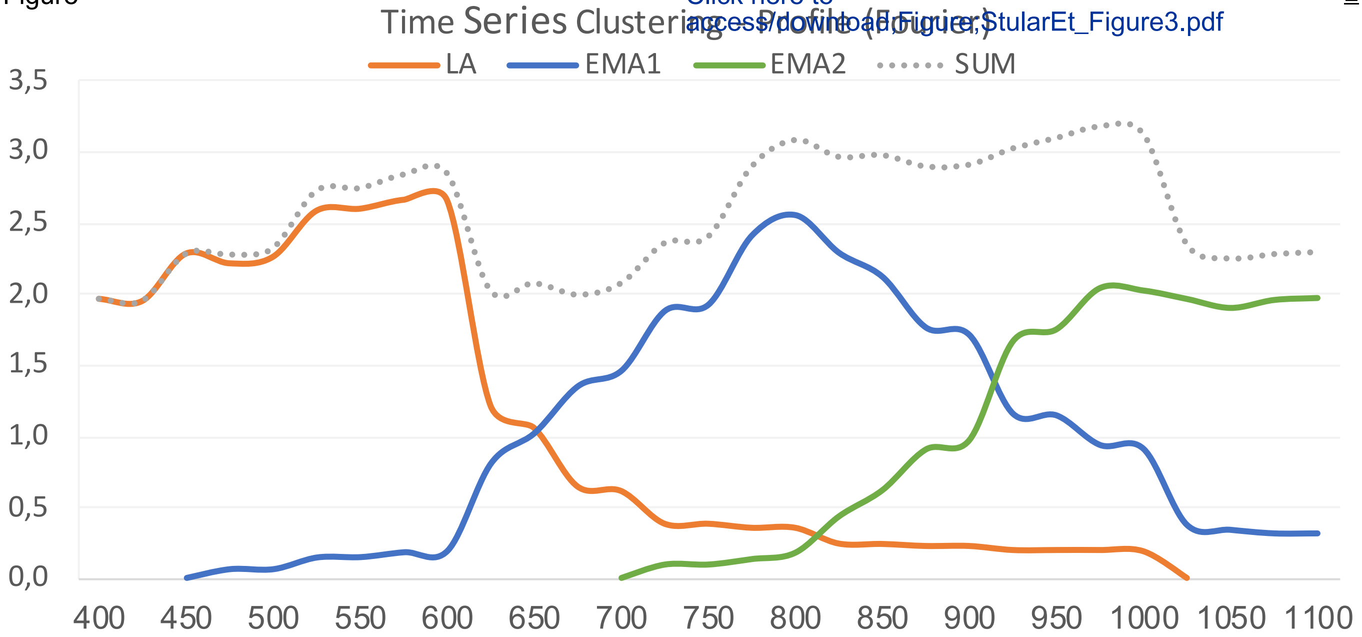

Figure

Click here to access/download;Figure;StularEt_Figure4.pdf

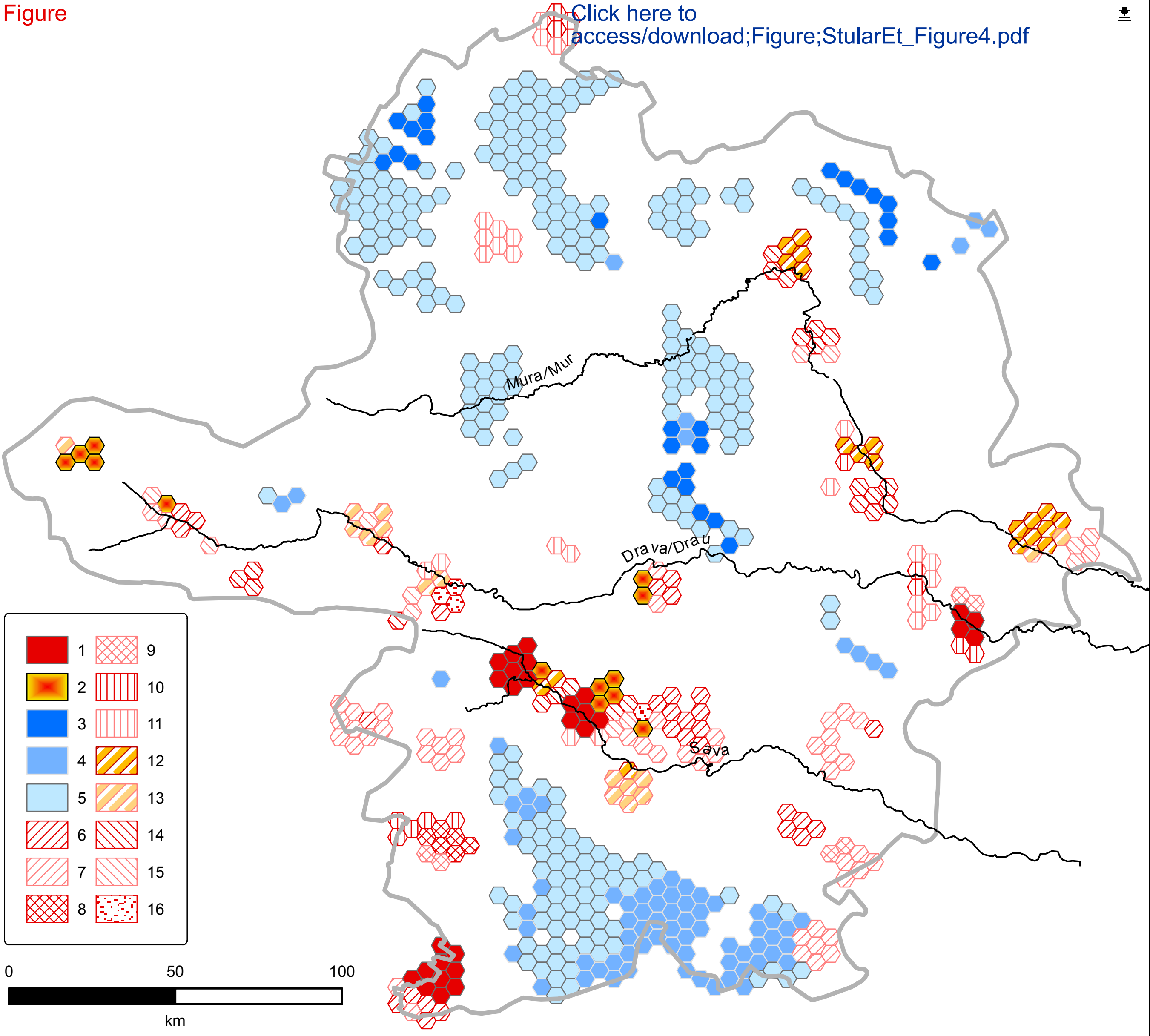

Click here to access/download;Figure;StularEt_Figure5.pdf

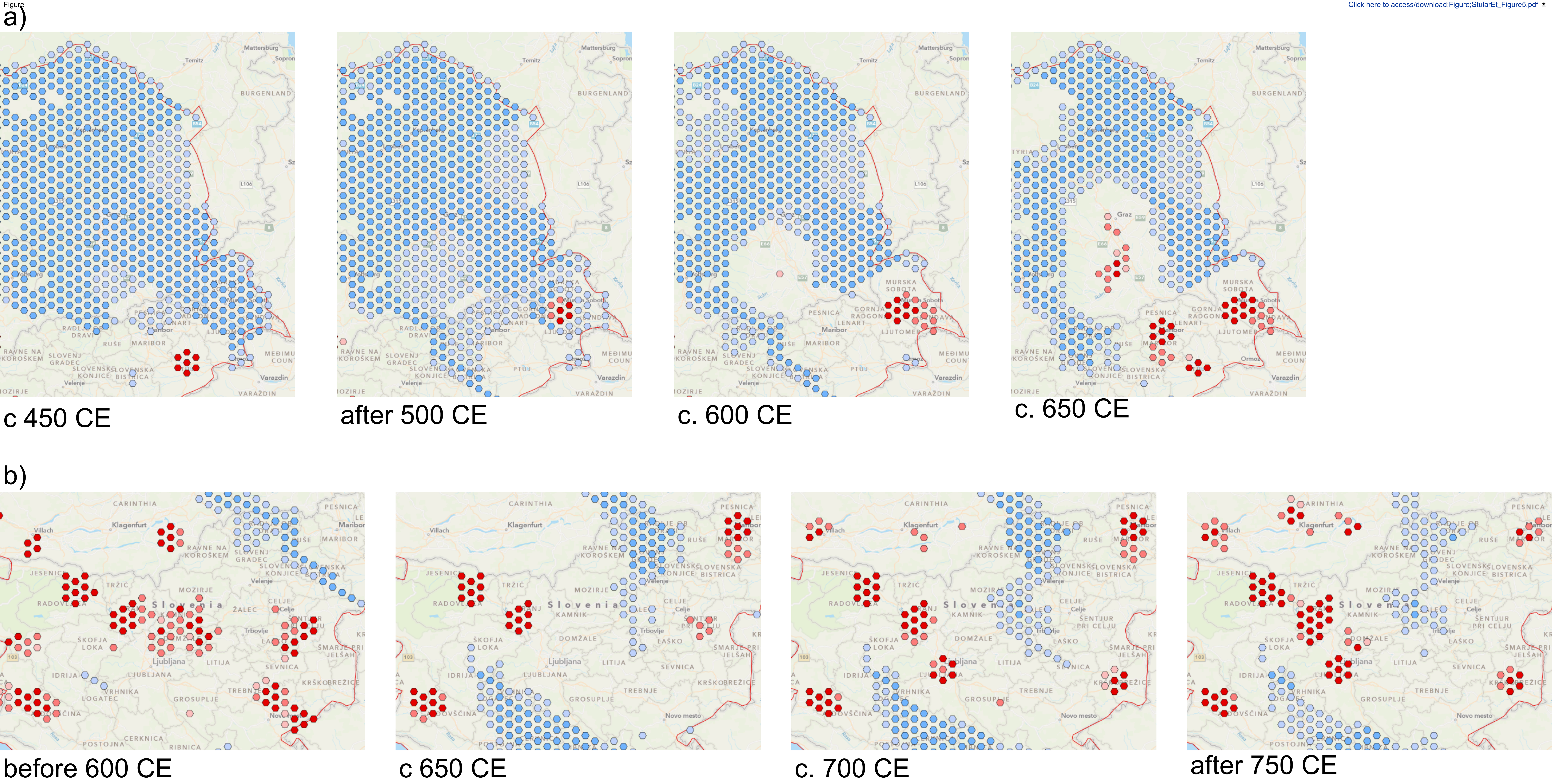

S1_Table

Click here to access/download
Supporting Information
StularEt_S1_Table.docx

Click here to access/download
Supporting Information
StularEt_S2_Appendix.docx

S3_Appendix

Click here to access/download

Supporting Information

StularEt_S3_Appendix_small.gif
