# Supplementary material for: Migration of Alpine Slavs and machine learning: Space-time pattern mining of an archaeological data set
Source: PLoS One. 2022 Sep 19;17(9):e0274687. doi: 10.1371/journal.pone.0274687 (PMC9484688; doi:10.1371/journal.pone.0274687)
Supplement: Attachment — Submitted filename: RebutalLetter.docx [file pone.0274687.s005.docx]

PONE-D-22-00071

Migration of Alpine Slavs and machine learning: space-time pattern mining of an archaeological data set

PLOS ONE

**Rebuttal letter**

Dear Editor, dear reviewers

The authors would like to thank the reviewers for their thorough and thoughtful reviews. We have implemented all the suggested minor comments that helped us to improve the exposition of the article. We have also addressed reviewer 2's general comments by introducing a more balanced assessment of the strength of the linguistic evidence.

Below are our responses, point by point (reviewers remarks in grey, our responses in black).

Reviewer #1:

111-112: "Spread of Common Slavic" is a little awkward - "Common Slavic" usually refers to the more or less uniform construct established by the comparative method, but ancillary methods such as etymology and dialect geography have demonstrated that "Late Common Slavic" was dialectally heterogeneous. Perhaps say "Spread of Slavic" and denote a time period.

Corrected:

... the spread of Slavic in Early Middle Ages…

112: Perhaps consider a 4th reason: center-periphery phenomena. Peripheries typically preserve archaism better than centers. Slovene is on the southwestern periphery of the spread of Slavic.

Corrected:

114: so strong links > such strong links
Corrected.

117: Old Church Slavic (capitalize, as it is used as a proper name). Better would be "canonical Old Church Slavic" (= corpus of Cyrillo-Methodian tradition texts), which excludes the Monumenta Frisingensia (as you intend).
Corrected:

...canonical Old Church Slavic...

118: a gloss and/or explanation of župan would help the uninitiated reader.
Corrected:

... the oldest mention of a member of a specifically Slavic social elite, *župan*,

429-430: Consider also:
Kushniarevich, Alena and Kassian, Alexei, “Genetics and Slavic Languages”, in: Encyclopedia of Slavic Languages and Linguistics Online, Editor-in-Chief Marc L. Greenberg. Consulted online on 11 April 2022 
First published online: 2020
Corrected:

The reference has been added.

432: "West Croats" is not clear: coastal (= Čakavian)? NW (= Kajkavian)? Note also that "White Croats" (Slovak: Bieli Chorváti) are identified in the medieval West Slavic region. Nobody has established who the "White Croats" were -- only the name is known and there is no evidence specifically linking them with (or denying linkage with) the South Slavic Croats.

Corrected:

It shows that the inhabitants of present-day Slovenia (and partly those from western Croatia) are far removed from all other South Slavic populations.

Reviewer #2

I find the topic of the study very interesting. However, I am not convinced that the linguistic evidence referred to by the authors is strong enough to support the conclusions they draw.
Accordingly, I would encourage the authors to either modify their confidence in their analysis of the linguistic evidence or to leave out entirely the linguistic component of the paper. I am aware that especially the latter option will make the paper less attractive as the linguistic perspective is important for the overall interest of the study.
An alternative, and in my opinion much more satisfying, solution would be to strengthen the linguistic dimension of the study. Instead of the vague and sporadic references to secondary literature (often in Slovenian, which – sadly – makes it accessible to only a minority of the readers of the journal), it would be interesting to see actual analyses of the linguistic evidence that the authors claim to have for their conclusions. This would probably require that a person with a thorough knowledge of the linguistic background be included in the group of authors, if such a person is not already present.
The authors have decided to modify our confidence in the interpretations of the linguistic evidence to which we refer in our work.

In addition, we have:

- expanded and substantiated our presentation of the cited linguistic interpretations;

- in the interests of a balanced article, we have also expanded and substantiated our presentation of the genetic history evidence;

- key interpretations from linguistics and genetics have been replaced by direct quotations to make it clearer that we are only using existing interpretations by domain specialists.

In the attached pdf file containing the study I have added my comments on specific parts. The file also includes corrections and stylistic suggestions.

We thank the reviewer for corrections and stylistic suggestions; we have implemented them in full, so there are now stylistic changes throughout the text.

Below are rebutals to the inline comments, listed by lines.

l.59: Corrected.

l.85: Corrected.

l.107: Corrected.

l.110-119: The entire paragraph has been rewritten to address the reviewer's comments. Especially with regard to the linguistic evidence, we have followed the recommendation of reviewer 1. It now reads:

“Compared to the entirety of Slavic territories ours is a small study region. But this region is an excellent (if not pivotal) case study for understanding the general processes of the spread of Slavic speakers in Early Middle Ages for three reasons. (i) Archaeologically, this is the only region where data is readily available for advanced spatial analysis, including machine learning (see above). (ii) The historiographical sources are second to none and include the oldest permanent Slavic political entity (Carantania, after 650 CE; e.g., [4,22,34-36]), the oldest Slavic text other than the canonical Old Church Slavic (ninth-century *Monumenta Frisingensia*; [37]), and the oldest mention of a member of a specifically Slavic social elite, a *župan* (iopan Physso, 777 CE [21,22,38]). (iii) Linguistically, the area is on the southwestern periphery of the spread of Slavic, bordering Germanic and Romance languages; this is important because peripheries typically preserve archaisms better than centres.«

l.156: Corrected.

l.262: Corrected, it now reads: »Moreover, this topic is invariably interdisciplinary, but this interdisciplinarity is achieved through a variety of approaches.«.

l.276: Corrected.

l.282: Corrected.

l.320: Corrected.

l.390-406: The entire subsection 5.2 has been rewritten and expanded to accomodate the reviewers comments. It now reads:

“Let us first take a look at linguistics. Linguistics holds that, while a language or dialect may be tied to any number of identities within a given period, a common linguistic innovation requires the community in which it occurs, the so-called founder population [6].

Modern Slovenian, which is spoken today in the southern part of the research area, belongs to the South Slavic Clade, according to the traditional classification of the Slavic languages [82]. There are, however, considerable linguistic similarities between the Slovenian and the West Slavic lects. These similarities were explained either by the existence of a specific link between Slovenian and West Slavic or by the mixed South and West Slavic origin of Slovenian [83-87].

Specific ties between Slovenian and South Slavic on the one hand and West Slavic on the other have recently been demonstrated with a series of phylogenetic NeighborNet networks. The analysis concluded that Slovenian seems to be almost equally close to the West and South Slavic, but distant from the East Slavic, "thus supporting the putative mixed nature of Modern Slovenian" [33]. It is the latter interpretation that interests us.

In conclusion of the above cited analysis further studies of Slovenian dialects are proposed in order to clarify the position of Slovenian among the Slavic languages. One of such study examined the diatopic distribution and semantic development of *gъlčěti as the primary neutral verb meaning 'to speak'. It was carried from an emergent dialect of Proto-Slavic and is now widespread in present-day central Russia, central Bulgaria, and in Slovenia along the Mura and Drava rivers. Of interest is the hypothesis of possible relationships between the "early Slavic speakers who spoke dialects in which *gъlčěti played a central role as a verb of speech" and those who did not within modern Slovenia, i.e. southern part of our study area. The hypothesis states that this dichotomy, together with the -ny- || -nǫ- isogloss, "can be viewed as inherited pre-migration cleavage" [54], that is, "the dialects of Slavic brought to the subalpine area ... differed (amongst themselves)" ([6]; translated from the Slovenian by B.Š.; the subalpine area mentioned corresponds approximately to our study region). Since "shared linguistic innovation presupposes a community" ([6]; translated from the Slovenian by B.Š.), it follows that heterogeneous dialects presuppose heterogeneous communities or founder populations.

Therefore, the linguistic interpretations imply that the southern part of the region under study was originally populated by two founder populations that spoke two heterogeneous Slavic dialects. One, using *gъlčěti, populated areas along the rivers Mura and Drava, the other one populated areas further west.”

l.441: Corrected.

l.446: Corrected, it now reads: »Archaeology, linguistics, and population genetics have each deduced with varying degrees of certainty that there were…«.

l.450-1: Corrected

l.450-2: The statement in the text is: »...linguistics confirms that the migrants were speakers of Common Slavic, ...« Reviewers comment is: »this is an overstatement«. Our statement is based on the text in 5.2 (now expanded) with references. To our knowledge, there are no scientific publications stating the contrary.

We therefore believe to have met the reviewers criteria by expanding the section 5.2.

l.455: See comment above.

l.458: See comment above.

l.460: Corrected.

l.477: Corrected.
