# Supplementary material for: Migration of Alpine Slavs and machine learning: Space-time pattern mining of an archaeological data set
Source: PLoS One. 2022 Sep 19;17(9):e0274687. doi: 10.1371/journal.pone.0274687 (PMC9484688; doi:10.1371/journal.pone.0274687)
Supplement: Attachment — Submitted filename: StularEt_Rebuttal.docx [file pone.0274687.s007.docx]

PONE-D-22-00071

Migration of Alpine Slavs and machine learning: space-time pattern mining of an archaeological data set

PLOS ONE

**Rebuttal letter**

Dear Editor, dear reviewers

The authors would like to thank the reviewer #2 for their thorough and thoughtful reviews. We have implemented all the suggested comments in full.

Below are our revisions (before marked in grey, now in black).

line 402-404

Let us first take a look at linguistics. Linguistics holds that, while a language or dialect may be tied to any number of identities within a given period, a common linguistic innovation requires the community in which it occurs, the so-called founder population [6].

Let us first take a look at linguistics. While a language or dialect may be tied to any number of identities within a given period, a shared linguistic innovation requires a linguistic community, for which the term "founder population" has been proposed [6, 55].

Comment: the term "founder population" was proposed by Greenberg and the text has been changed to reflect this; also, the reference has been amended accordingly.

line 406

...the South Slavic Clade,...

...the South Slavic clade,...

line 409

... by the mixed South and West Slavic origin...

... by a mixed South and West Slavic origin...

line 422

... i.e. southern part...

... i.e. the southern part...

line 424

... pre-migration cleavage"...

... pre-migration cleavages"...

line 475-476

Archaeology, linguistics, and population genetics have each deduced with varying degrees of certainty that there were two separate migrations...

Archaeological, linguistic, and genetic evidence suggests, with varying degrees of certainty, that there were two separate migrations…

line 480-481

Linguistics and genetics have interpreted that the migrants were Slavs. In particular, linguistics confirms that the migrants were speakers of Slavic, and genetics confirms that they had a...

Linguistics and genetics indicate that the migrants were Slavs. In particular, linguistics indicates that the migrants were speakers of Slavic, and genetics confirms that they had a…

line 485

Table 1 has been removed.

line 606

Updated reference of an article that is now in press.
